# The Translational Medicine Ontology and Knowledge Base: driving personalized medicine by bridging the gap between bench and bedside

**DOI:** 10.1186/2041-1480-2-S2-S1

**Published:** 2011-05-17

**Authors:** Joanne S Luciano, Bosse Andersson, Colin Batchelor, Olivier Bodenreider, Tim Clark, Christine K Denney, Christopher Domarew, Thomas Gambet, Lee Harland, Anja Jentzsch, Vipul Kashyap, Peter Kos, Julia Kozlovsky, Timothy Lebo, Scott M Marshall, Jamie P McCusker, Deborah L McGuinness, Chimezie Ogbuji, Elgar Pichler, Robert L Powers, Eric Prud’hommeaux, Matthias Samwald, Lynn Schriml, Peter J Tonellato, Patricia L Whetzel, Jun Zhao, Susie Stephens, Michel Dumontier

**Affiliations:** 1 Rensselaer Polytechnic Institute, Troy, NY, USA; 2 Predictive Medicine Inc., Belmont, MA, USA; 3 AstraZeneca, Lund, Sweden; 4 Royal Society of Chemistry, Cambridge, UK; 5 National Library of Medicine, Bethesda, MD, USA; 6 Harvard Medical School, Boston, MA, USA; 7 University of Manchester, Manchester UK; 8 Eli Lilly and Company, Indianapolis, IN, USA; 9 Albany Medical Center, Albany, NY, USA; 10 W3C, Cambridge, MA, USA; 11 Pfizer, Sandwich, UK; 12 Freie Universität, Berlin, Germany; 13 Cigna, Hartford, CT, USA; 14 AstraZeneca, Waltham, MA, USA; 15 Leiden University Medical Center, Leiden, NL; 16 University of Amsterdam, Amsterdam, NL; 17 Case Western Reserve University School of Medicine, Cleveland, OH, USA; 18 W3C HCLSIG, W3C, Cambridge, MA, USA; 19 Medical University of Vienna, Vienna, Austria; 20 Information Retrieval Facility (IRF), Vienna, Austria; 21 Digital Enterprise Research Institute (DERI), National University of Ireland Galway, Ireland; 22 University of Maryland, Institute for Genome Sciences; 23 Stanford University, Stanford, CA, USA; 24 University of Oxford, Oxford, UK; 25 Johnson & Johnson Pharmaceutical Research & Development L.L.C., Radnor, PA, USA; 26 Carleton University, Ottawa, Canada

## Abstract

**Background:**

Translational medicine requires the integration of knowledge using heterogeneous data from health care to the life sciences. Here, we describe a collaborative effort to produce a prototype Translational Medicine Knowledge Base (TMKB) capable of answering questions relating to clinical practice and pharmaceutical drug discovery.

**Results:**

We developed the Translational Medicine Ontology (TMO) as a unifying ontology to integrate chemical, genomic and proteomic data with disease, treatment, and electronic health records. We demonstrate the use of Semantic Web technologies in the integration of patient and biomedical data, and reveal how such a knowledge base can aid physicians in providing tailored patient care and facilitate the recruitment of patients into active clinical trials. Thus, patients, physicians and researchers may explore the knowledge base to better understand therapeutic options, efficacy, and mechanisms of action.

**Conclusions:**

This work takes an important step in using Semantic Web technologies to facilitate integration of relevant, distributed, external sources and progress towards a computational platform to support personalized medicine.

**Availability:**

TMO can be downloaded from http://code.google.com/p/translationalmedicineontology and TMKB can be accessed at http://tm.semanticscience.org/sparql.

## Background

A major element of personalized medicine involves the identification of therapeutic regimes that are safe and effective for specific patients. This contrasts the “one-size-fits-all” well-known concept of “blockbuster” drugs, which are considered safe and effective for the entire population. The concept of targeted patient groups falls in-between these two extremes with the identification of therapeutic regimes targeted to be safe and effective for specific patient groups with similar characteristics [1]. A number of factors have contributed to a decline in the emphasis of blockbuster therapeutics and a corresponding rise in the quest for tailored therapeutics or personalized medicine. Essential to the realization of personalized medicine is the development of information systems capable of providing accurate and timely information about potentially complex relationships between individual patients, drugs, and tailored therapeutic options. The demands of personalized medicine include integrating knowledge across data repositories that have been developed for divergent uses, and do not normally adhere to a unified schema. This paper demonstrates the integration of such knowledge across multiple heterogeneous datasets. We show the formation of queries that span these datasets, connecting the information required to support the goal of personalized medicine from both the research and the clinical perspectives.

Integration of the patient electronic health record (EHR) with publicly accessible information creates new opportunities and challenges for clinical research and patient care. For example, one challenge is that the complexity of the information provided to the clinician must not impair the clinician’s ability to accurately and rapidly prescribe drugs that are safe and effective for a specific patient, and covered by the patient’s insurance provider. An example opportunity is that EHRs enable the identification of adverse events and outbreak awareness and provide a rich set of longitudinal data, from which researchers and clinicians can study disease, co-morbidity and treatment outcome. Moreover, the increased desire to rapidly translate drug and gene-based drug therapy to clinical practice depends on the comprehensive integration of the entire breadth of patient data to facilitate and evaluate drug development [2]. Thus, EHR integration could create the ideal conditions under which new or up-to-date evidence-based guidelines for disease diagnosis and treatment can emerge. Although supplying patient data to the scientific community presents both technical and social challenges [3], a comprehensive system that maintains individual privacy but provides a platform for the analysis of the full extent of patient data is vital for personalized treatment and objective prediction of drug response [4]. The impetus to collect and disseminate relevant patient-specific data for use by clinicians, researchers, and drug developers has never been stronger. Simultaneously the impetus to provide patient-specific data to patients in a manner that is accurate, timely, and understandable, has also never been stronger.

This motivation takes specific form in the US where health care providers who want stimulus-funded reimbursement from recent electronic health funding, to implement or expand the use of electronic medical records (EMRs) in care practices, must achieve “meaningful use.” An EMR is an electronic record of health-related information on an individual that is created, gathered, managed, and consulted by licensed clinicians and staff from a single organization who are involved in the individual’s health and care. An electronic health record (EHR) is an aggregate electronic record of health-related information on an individual that is created and gathered cumulatively across more than one health care organization and is managed and consulted by licensed clinicians and staff involved in the individual’s health and care. By these definitions, an EHR is an EMR with interoperability (i.e. integration to other providers’ systems). Achieving meaningful use requires both using certified EHR technology and achieving documented objectives that improve the quality, safety, and efficiency of care while simultaneously reducing disparities, engaging patients and families in their care, promoting public and population health, improving care coordination, and promoting the privacy and security of EHRs (CMS 2010) [5]. A “certified” EHR must meet a collection of regulations and technical requirements to perform the required meaningful use functions (ONCHIT 2010) [6]. Minimum meaningful use requirements include fourteen core objectives, five out of ten specific objectives, and fifteen clinical quality measures (CMS 2010). These criteria, conditions, and metric achievements are all delayed and complicated by the typical data fragmentation that occurs between the research and health care settings and will continue until a “translational” ontology is available to bridge activities, transferring data and entities between research and medical systems.

Translational medicine refers to the process by which the results of research done in the laboratory are directly used to develop new ways to treat patients. It depends on the comprehensive integration of the entire breadth of patient data with basic life science data to facilitate and evaluate drug development [2]. In the 1990s, several efforts related to data integration emerged, including the Archimedes Project and the use of heterogeneous data integration, mathematical and computational modeling, and simulation to expose the underlying dynamics and different individual treatment response patterns clinicians observed in patients diagnosed with Major Depressive Disorder [7][8]. When information regarding the patient experience (symptoms, pharmacokinetics/pharmacodynamics, outcomes, side effects) can be directly linked to biomedical knowledge (genetics, pathways, enzymes, chemicals, brain region activity), clinical research can gain new insights in causality and potential treatments. Detailed recordings of clinical encounters are a crucial component of this approach [9][10] and devices such as personal electronic diaries aid both patient and clinician in capturing accurate patient data of these accounts.

Electronic Medical Records now act as main repositories for patient data. As we continue to explore the intricate relationship between phenotype and genotype, these records become a vital source for monitoring patients’ progression of disease. The presence of a given variation, as it relates to the appearance or absence of disease over time, can be mapped as encounters are recorded by clinicians. Every result, encounter, event, or diagnosis is recorded as a data item and includes a date. This rich longitudinal data provide trends that show improvement or decline in state and occurrence or absence of diagnostic criteria and can be used to guide treatment, provide prognosis, or identify patients who are likely to respond to a potential treatment. The following example illustrates the kinds of data we seek to integrate and analyze for clinical research purposes. Carvedilol is prescribed to a given patient, while a number of blood pressures and heart rate recordings are taken sequentially over time. If this patient takes the medication as prescribed, we can easily observe trends and establish alerts to adjust the medication, if necessary. Alternatively, the simultaneous occurrence of any recorded side effects can be correlated more easily with potential causative agents. Increases or decreases in laboratory parameters can also be viewed graphically and displayed for easy review by clinicians. Rich longitudinal data can also provide the opportunity to validate diagnostic procedures and otherwise catch discrepancies between corresponding clinical reports. This application of longitudinal data is being investigated in the World Wide Web Consortium (W3C) Health Care and Life Science Interest Group (HCLSIG) within the context of breast cancer, where a radiology report is followed by a biopsy and a pathology report. There should be a set of corresponding observations within the two reports, with the pathology report corroborating the findings of the radiology report [11].

Semantic Web technologies enable the integration of heterogeneous data using explicit semantics, the expression of rich and well-defined models for data aggregation, and the application of logic to gain new knowledge from the raw data [12]. Semantic technologies can be used to encode metadata such as provenance, i.e. the original source where the data came from and how it was generated [13][14]. There are four main Semantic Web standards for knowledge representation: Resource Description Framework (RDF), RDF Schema (RDFS), Web Ontology Language (OWL), and SPARQL query language.

Ontologies, which formalize the meaning of terms used in discourse, are expected to play a major role in the automated integration of patient data with relevant information to support basic discovery and clinical research, drug formulation, and drug evaluation through clinical trials. Already, OWL ontologies have been developed to support drug, pharmacogenomics and clinical trials [15][16][17], provide a mechanism for the integration and exchange of biological pathways [18,19], and are increasingly being used in health care and life sciences applications [20]. Another W3C standard, Gleaning Resource Descriptions from Dialects of Languages (GRDDL) enables users to obtain RDF triples out of XML documents. Collectively, these next generation Semantic Web technologies provide the resources required to systematically re-engineer both EHR and research data warehouse systems. This will make it easier and more practical to integrate, query, and analyze the full spectrum of relevant laboratory and clinical research data, as well as EHRs, in supporting the development of cost effective and outcome-oriented systems.

In this paper, participants in the Translational Medicine task force of the World Wide Web Consortium’s Health Care and Life Sciences Interest Group (W3C HCLSIG) present the Translational Medicine Ontology (TMO) and the Translational Medicine Knowledge Base (TMKB). The TMKB consists of the TMO, mappings to other terminologies and ontologies, and data in RDF format spanning discovery research and drug development, which are of therapeutic relevance to clinical research and clinical practice. The TMO provides a foundation for types declared in Linking Open Drug Data (LODD) [21] and EHRs. The TMO captures core, high-level terminology to bridge existing open domain ontologies and provides a framework to relate and integrate patient-centric data across the knowledge gap from bench to bedside. With the TMO and TMKB, we demonstrate how to bridge the gap and how to develop valuable translational knowledge pertinent to clinical research, and therefore to clinical practice.

The remainder of the paper is structured as follows: we describe the use case for the TMKB, which centers around Alzheimer’s Disease (AD), then describe the methods used to build the TMKB, the ontology design process, data sources, and mappings. We then explore pertinent questions that the TMKB can answer in the results, discuss our findings, and conclude with a listing of unsolved problems and possible future directions for this work.

### Use case

Alzheimer’s Disease (AD) is an incurable, degenerative, and terminal disease with few therapeutic options [22][23]. It is a complex disease influenced by a range of genetic, environmental, and other factors [23]. Recently, Jack *et al.*[24] demonstrated the value of shared data in AD biomarker research. A New York Times article on the role of data sharing, in the advancement of AD research, quotes John Trojanowski at the University of Pennsylvania Medical School: “It’s not science the way most of us have practiced it in our careers. But we all realized that we would never get biomarkers unless all of us parked our egos and intellectual-property noses outside the door and agreed that all of our data would be public immediately.” [25] Efficient aggregation of relevant information improves our understanding of disease and significantly benefits researchers, clinicians, patients and pharmaceutical companies.

We demonstrate the usefulness of TMO and TMKB in a use case that follows a patient and physician from a first report of symptoms, to diagnosis of AD, selection of an optimal treatment regimen, consideration of alternative treatments following the report of side effects caused by the initial treatment, and finally to the selection of possible appropriate clinical trials for the patient.

The Alzheimer’s Disease patient use case can be summarized in the following way:

1. A patient and family members report symptoms to a physician/clinician. The physician/clinician enters the reported symptoms into an EHR. All concepts are mapped to URIs with the help of TMO.

2. The physician makes a list of differential diagnoses, with a working diagnosis of AD.

3. The physician arranges for the patient to have a basic biochemical, haematological, and SNP profile undertaken. Biochemistry, haematology, and SNP requests are input directly by the various respective departments into the patient’s EHR. Preliminary SNP and genetic data will be submitted directly to the NIH Pharmacogenetics Research Network (PGRN).

4. A follow-up meeting is scheduled to perform a set of diagnostic tests outlined by what the clinician feels initially are most appropriate for disease presentation.

5. The physician continues to add investigations/lab results to the patient’s EHR and these are combined with the patient’s medical history information. A disease is chosen as the most likely of the listed differential diagnoses based on all of the information provided.

6. The physician confirms and now has a refined and widely acceptable diagnosis of AD with behavioral assessments, cognitive tests, and appropriate brain scan if indicated and enters the diagnosis data into the patient’s EHR.

7. The physician selects the most appropriate AD drug and clinical protocol from the patient’s medical record based on the severity of the disease, the patient’s SNP profile (ADME, efficacy/safety based on presence or absence of receptors), patient’s BMI, and concurrent medication, and drug availability on Medicare D. Fundamental questions will be answered by the ontology at this stage by sourcing relevant data sets simultaneously or in a specific order:

• *What are the clinically recommended agents?*

• *What products are available for prescription, and which are legally indicated for AD disease?*

• *What is the SNP verdict? These agents are sourced with a pharmacogenomics database to determine*

– *Will they be efficacious? Is the disease receptor positive?*

– *Will they be harmful? Are there toxic metabolites? Is CYP 450 or acetylator status available?*

• *Are the preceding predictive genetic SNP tests covered by the patient’s insurance company? Are the resulting pharmaceutical agents covered by the patient’s specific insurance?*

8. The physician checks with the pharmacist, or consults drug information literature to avoid potential drug interactions.

9. The physician now prescribes Aricept (Donepezil) as it satisfies criteria listed above. It is indicated, safe, effective, available, there are no drug interactions issues with drug delivery, and it is covered by the insurance.

10. In a follow-up visit the patient later reports nausea from Donepezil. The physician is aware of this common side effect (other side effects reported include bradycardia, diarrhea, anorexia, abdominal pain, and vivid dreams etc...), and re-consults the literature to ensure this is acceptable and agreeable with patient. The physician documents the side effect for post-marketing adverse event pick-up and future study. He changes medication if necessary or adds another medication to alleviate side effects.

11. The physician considers moving the patient to a trial. The physician obtains information on all (local, national, and international trials) for AD. Trials might be listed in data sources from the FDA, WHO, ClinicalTrials.gov, Citeline TrialTrove, etc.; academia or pharma may also solicit patients, or the physician may point the patient to investigators undertaking a trial.

• *The physician decides whether*

– *to enroll the patient in a clinical trial as one of the agents looks very suitable and may benefit patient, or because the patient is interested in participating in the trial;*

– *not to enroll the patient because the trial is unsuitable or the patient declines to participate in the trial;*

– *to obtain information for the patient on a trial appropriate for the patient with potential of future enrollment.*

12. The physician checks if the patient meets trial inclusion/exclusion criteria by querying the EHR.

13. The patient has a thorough medical assessment (lifestyle, medical history, genomics, proteomics, metabolomics, images, cognition) to supplement and update existing data.

14. The results of the medical exam influence the arm of the trial in which the patient participates. The patient status is updated.

Questions relevant for this use case scenario are listed in Table 1. Such questions can be formulated in SPARQL queries (see section SPARQL queries, and additional file 1) and answered using TMKB.

**Table 1:** Questions and answers using TMO-integrated data sources

Question	Answer
*Clinic*	
What are the diagnostic criteria for AD?	There are 12 diagnostic inclusion criteria and 9 exclusion criteria.
Does Medicare D cover Donepezil?	Medicare D covers 2 brand name formulations of Donepezil: Aricept and Aricept ODT.
Have any AD patients been treated for other neurological conditions?	Patient 2 was found to suffer from AD and depression.
*Clinical Trial*	
Since my patient is suffering from drug-induced side effects for AD treatment, can an AD clinical trial with a different mechanism of action (MOA) be identified?	Of the 438 drugs linked to AD trials, only 58 are in active trials and only 2 (Doxorubicin and IL-2) have a documented MOA. 78 AD-associated drugs have an established MOA.
Find AD patients without the APOE4 allele as these would be good candidates for the clinical trial involving Bapineuzumab?	Of the four patients with AD, only one does not carry the APOE4 allele, and may be a good candidate for the clinical trial.
What active trials are ongoing that would be a good fit for Patient 2?	58 Alzheimer trials: 2 mild cognitive impairment, 1 hypercholesterolaemia, 66 my-ocardial infarction, 46 anxiety, and 126 depression.
*Research*	
What genes are associated with or implicated in AD?	Diseasome and PharmGKB indicate at least 97 genes have some association with AD.
Which SNPs may be potential AD biomark-ers?	PharmGKB reveals 63 SNPs
Which market drugs might potentially be re-purposed for AD because they modulate AD implicated genes?	57 compounds or classes of compounds that are used to treat 45 diseases, including AD, hyper/hypotension, diabetes and obesity.

## Methods

Please refer to the public wiki page for specific URLs of resources described herein [26]. As part of its requirements analysis, the HCLSIG Translational Medicine task force identified seven use cases against which its activities would be measured. These include scenarios involving chemogenomics, animal models, pharmacogenomics, therapeutic development, patient care, and integrative informatics (see wiki for full details of each use case). The work presented here follows questions asked in the patient care scenario that are related to the user roles and interests summarized in Table 2.

**Table 2:** Users and their interests in translational medicine

Category	User	Interest
Research	Biologist (*in vivo*, *in vitro*, *cellular & molecular*)	Target identification, assay development, target validation
	Bioinformatician	Biological knowledge management, cellular modeling
	Immunologist	Natural defense mechanisms
	Cheminformatician	Predictive chemistry
	Medicinal chemist	Drug efficacy
	Systems physiologist	Tolerance, adverse events
Clinic	Clinical trial specialist	Trial formulation, recruitment
	Clinical decision support	Data analysis, trend finding
	Primary care physician	General, conventional care
	Specialty medical provider	Specialized treatments
Business	Sales & marketing	Revenue generation
	Strategic/portfolio manager	Assessing market opportunities
	Project manager	Prioritizing resources & activities
	Health plan provider	Insurance coverage

We present the major components of the TMKB, namely the ontology used as a framework for data integration and the various datasets integrated in our knowledge base. We also outline the processes that we developed for ensuring the consistency of the knowledge base and the ontology.

### Ontology design

The scope of the Translational Medicine Ontology (TMO) is defined by the use case terminology and respective data sources. Each term and corresponding data source was analyzed for its conceptual, representational and reasoning capability as required by the use case requirements. TMO terms were obtained from a lexical analysis of sample research questions from 14 types of users, all of whom were involved in aspects of research, clinical care and or business (Table 2). Terms were formalized as referring to classes, relations or individuals in the OWL ontology. Terms that appear in statements that hold in general (e.g. “patients participate in consultations” and “active ingredient is a role played by a molecular entity”) form key background knowledge, refer to instantiable types and are represented as classes in the ontology. Eighty classes were created to represent material (e.g. molecule, protein, cell lines, pharmaceutical preparations), processual (e.g. diagnosis, study, intervention), qualitative, role (e.g. subject, target, active ingredient) and informational entities (e.g. dosage, mechanism of action, sign/symptom [27], family history) of relevance to our study. By contrast, particulars (e.g. “a patient with a given name” and “a blister package of a pharmaceutical product with a particular identifying code on it”) refer to individuals and are represented as instances of classes in the ontology. Consequently, a particular consultation at a given time and day, the particular patient role in that consultation, and the physician role in that consultation can be represented as instances of classes in the ontology.

**Figure 1:**
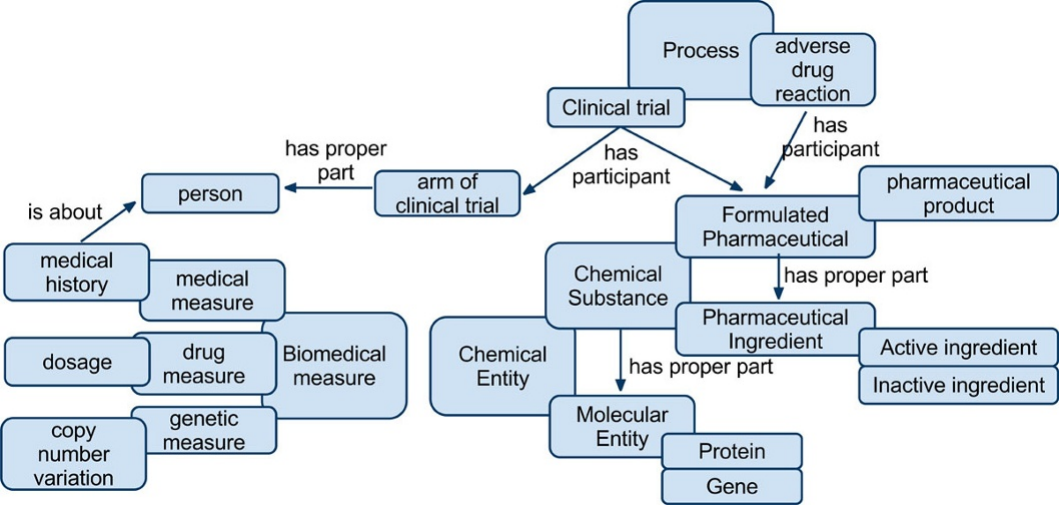
**TMO overview.** Overview of selected types, subtypes (overlap) and existential restrictions (arrows) in the Translational Medicine Ontology.

**Figure 2:**
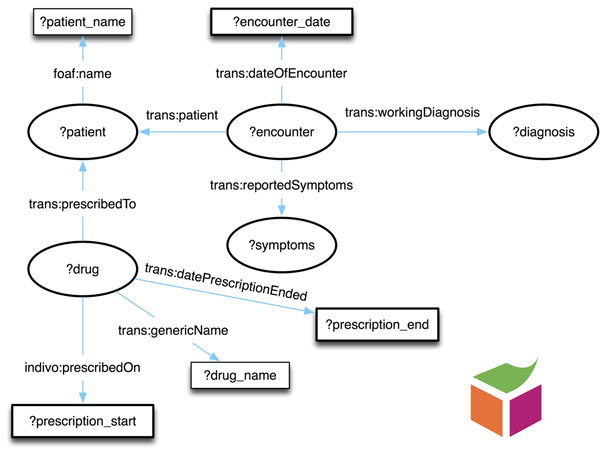
**Query #1: Side effects.** The data elements involved in query #1. The query can be formulated as “How many patients experienced side effects while taking Donepezil?”

**Figure 3:**
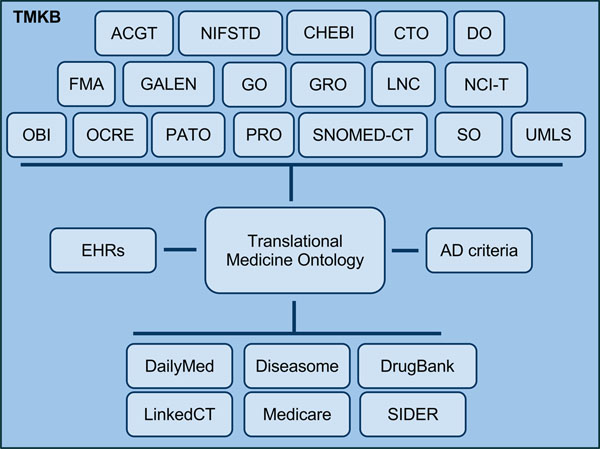
**TKMB overview.** Overview of the contents of the Translational Medicine Knowledge Base (TMKB). TMKB is composed of the Translational Medicine Ontology with mappings to ontologies and terminologies listed in the NCBO BioPortal. The TMO provides a global schema for Indivo-based electronic health records (EHRs) and can be used with formalized criteria for Alzheimer’s Disease. The TMO maps types from Linking Open Data sources.

Figure 1 shows a portion of the TMO and illustrates selected types, subtypes, and existential restrictions that hold between types. For instance, chemical substances are chemical entities that are composed of molecular entities. A key part of designing the ontology involved disambiguating polysemous terms e.g. “drug.” A drug can refer to the whole pharmaceutical product or to the active ingredient. The TMO differentiates these meanings as a “molecular entity” (TMO 0034) for individual molecules, “active ingredient” (TMO _0000) for biologically active chemicals in formulated pharmaceuticals, “formulated pharmaceutical” (TMO _0001) for a substance that may or may not have been approved by a regulatory authority, and “pharmaceutical product” (TMO _0002) for a drug approved by a regulatory authority. The TMO extends the basic types defined in the Basic Formal Ontology and uses relations from the Relation Ontology [28]. Given the prevalence of the terms defined in the ontology and the desire to establish the TMO as a global ontology, we also created 223 class equivalence mappings (using *owl:equivalentClass*) from 60 TMO classes to 201 target classes from 40 ontologies (see Table 3; Figures 2 and 3). These mappings were manually identified and verified using the NCBO BioPortal [29] and UMLS [30]. Finally, in order to create a stable, consistent ontology, we import one document (TMO-external.owl) as the aggregation of all externally dependent ontologies, including: Basic Formal Ontology (BFO), Relation Ontology (RO), and Information Artifact Ontology (IAO).

**Table 3:** Representative mappings between TMO and target terms

Label	TMO	Target
Protein	0035	ACGT:Protein, BIRNLex:23, CHEBI:36O8O, FMA:Protein, GO:OOO3675, GRO:Protein, Galen:Protein, NCIt:Protein, PRO:00000000l, SNOMEDCT:88878007, SO:0000358, UMLS:C0033684
Gene	0037	FMA:Structural gene, GRO:Gene, Galen:Gene, LNC:LP32747-5, MSH:D005796, NCIt:Gene, NCIt:Gene_ Object, NDFRT:C242394, PRO:Gene, SNOMEDCT:6727l00l, SO:0000704, UMLS:C00I7337
Diagnosis	0031	ACGT:Diagnosis, FHHO:Diagnosis, Galen:Diagnosis, LNC:LP72437-4, MSH:D003933, NCIt:Diagnosis, OBI:0000075, OCRe_clinical:Diagnosis, SNOMEDCT:439401001, UMLS:C0011900
Disease	0047	ACGT:Disease, BIRNLex:ll0l3, DOID:4, GRO:Disease, LNC:LP21006-9, MSH:D004194, NCIt:Disease_ or_ Disorder, NDFRT:C2140, OBI:0000155, UMLS:C0012634

Abbreviations: ACGT- ACGT Master Ontology, NIFSTD – Neuroscience Information Framework Standardized ontology, CHEBI – Chemical Entities of Biological Interest, CTO – Clinical Trial Ontology, DOID – Human Disease Ontology, FMA – Foundation Model of Anatomy, FHHO – Family Health History Ontology, Galen – Galen Ontology, GO – Gene Ontology, GRO – Gene Regulation Ontology, LNC – Logical Observation Identifier Names and Codes, MSH- Medical Subject Headings, NCIt – NCI theraurus, NDFRT – National Drug File, OBI – Ontology for Biomedical Investigation, OCRe - Ontology for Clinical Research, PATO – Phenotypic Quality Ontology, PRO – Protein Ontology, SNOMED CT, SNOMED clinical terms, SO – Sequence Ontology, UMLS – Unified Modeling Language System.

The TMO was built using Protégé 4.0.2 and is represented as an OWL2 compliant ontology. TMO Terms are defined in the http://www.w3.org/2001/sw/hcls/ns/transmed/ namespace. See public wiki to obtain the ontology.

### Data sources

The data sources used in this study include formulary lists, pharmacogenomics information, clinical trial lists, and scientific data about marketed drugs (Table 4). ClinicalTrials.gov is a registry of clinical trials, AD diagnostic refers to a formalized version of the diagnostic criteria for Alzheimer’s Disease described in Dubois et al. [31] , DailyMed contains marketed and FDA approved drugs, Diseasome contains information about gene-disease associations, DrugBank [32] contains detailed drug and drug target data, Medicare contains Medicare Part D approved drugs, Patient contains the synthetic patient data created for use in this study, PharmGKB [33] contains data about drug response associated with genetic variation and SIDER identifies side effects associated with marketed drugs.

**Table 4:** Data sources used in this study

LODD	Prefix	Dataset	Description
x	linkedct	Clinicaltrials.gov	Registry of clinical trials
	dubois	AD diagnostic	AD diagnostic criteria
x	dailymed	DailyMed	Marketed & FDA approved drugs
x	diseasome	Diseasome	The genetic basis of disease
x	drugbank	DrugBank	Detailed drug data & drug target
x	medicare	Medicare	Medicare D approved drugs
	pchr	Patient	synthetic patient data
	pharmgkb	PharmGKB	Drug response to genetic variation
x	sider	SIDER	Side effects of marketed drugs

LODD – ‘x’ indicates a Linking Open Drug Data dataset

All datasets, except for PharmGKB, diagnostic criteria, and patient records, are available through the LODD project [21]. PharmGKB is made available as part of the Bio2RDF project [34]. URLs for the data sources are available on the Translational Medicine wiki. [26] Seven synthetic patient records were manually created to capture typical medical record data: demographic information, contact information, family history, life style data, allergies, immunizations, information on conditions, procedures, prescriptions, and visits to health care providers. These records are by no means complete, or unabridged. In practice, clinicians often base care on similar records when treating patients. Patients typically seen by care providers in one health care network, using one EHR system, may visit another hospital outside their network that uses a completely different EHR system. This results in an unfortunate, but common real-world scenario that forces the creation of a duplicate EHR, often simplistic and based largely on the data contained in the previous system. In many instances, crucial information is transferred via telephone to the new provider because alternative means are often not yet in place to enable electronic transfer or interoperability in a timely fashion. In this way, the patients created for the TMO reflect the type of health record one could expect to see in clinical practice. They are basic, yet contain enough data to demonstrate a foundation for more complex query as standardized systems become more prevalent.

The United States Department of Veterans Affairs currently maintains one of the most comprehensive EHR repositories, the Computerized Patient Record System (CPRS), which is managed with the related clinical VistA software. A patient’s medical record within this system will likely contain far more detail than the simulated patients we have created for TMO. If a patient visits any facility within the Veteran’s Network, a complete unabridged medical record is fully accessible and may be updated by all who access this record. Often times, patients never leave this network and all of their details remain in one profile. If the patient chooses to receive care at a hospital outside this network, as described above, only relevant details pertaining to their care are transferred. A new, abridged EHR is then re-created at each new institution, in much less detail, and is largely similar to the simulated patients designed for TMO.

Our records were, to a large extent, built upon the XML-based Indivo specification for personally-controlled health care records. The Indivo initiative [35] offers simple user interfaces to store records and to grant others controlled access to them. Archiving systems like i2b2’s database records and Indivo’s XML records can generically record data, such as test results, in tuples that include a coding system, a code, a tested value, and the units of the value. For example, a systolic blood pressure measurement might be listed using a SNOMED CT code and mmHg units as in the example below:

We used GRDDL/XSLT to define an RDF representation for Indivo patient records. A straightforward RDF representation of the above XML is:

Where possible, this representation instantiates types in the TMO ontology. However, this representation leaves the consumer having to normalize (e.g. MPa to mmHg) before comparing or reporting values of potentially different units. Representing frequently needed and commonly used vital signs in a normalized form simplifies the effort needed to reuse these data:

Including the generic and the “standardized” forms allows us to meet a wide range of use cases and the tension between flexibility and predictability is the crux of the art of standards.

Given that an XSLT stylesheet converts the XML-based Indivo data to instances of TMO classes, the mapping process should also perform this normalization. Currently, we normalize only a small set of vitals as a proof of concept, but this is expected to expand as we draw on more diverse data.

### Incremental-test-driven development

In order to keep our queries synchronized with the data model, we developed a simple test mechanism based on a practice of incremental development and testing. When changes are made to the data, incremental testing provides an efficient way to test all the known queries that area impacted by the changes. Practically, this means critiquing the accuracy of the RDF representation, deciding whether it should be modeled differently, making changes (in our case, to the XSLTs which generate the RDF), and finally invoking the unit testing system to determine whether queries can still be answered. The advantages of this workflow are increased accountability, increased agility/confidence, and error messages tied to recent edits. Our testing strategy could be described as “Extreme Ontology Development” akin to a programming methodology called “Extreme Programming” which incorporates regular and automated testing of essential application features into the development cycle and increases vigilance to the inadvertent errors that are typically introduced during development.

### Data mapping

The user roles and interests listed in Table 2 are related to the patient scenario use case described in 14 steps above and in greater detail on the public wiki. The first step in mapping was to work through each step of the Patient Scenario, identifying key terms and a standard ontology that contains that term. In the absence of identical matches on the labels, the Linkage Query Writer (LinQuer) tool was used to create mappings between LODD datasets [36], along with Silk [37], which employs similarity metrics including string, numeric, data, URI, and set comparison methods. Entity identity was asserted using *owl:sameAs*. The mappings were augmented by those provided for PharmGKB via Bio2RDF [34]. Mappings between LODD dataset types and the TMO types were established using *owl:equivalentClass*.

### TMKB

The TMKB is an RDFS-reasoning-capable Semantic Web knowledge base composed of the TMO, RDFized datasets, and equivalence mappings (Figure 3). The TMO, dataset, and mapping files were loaded into OpenLink Virtuoso 6 open source community edition, and is made available as a SPARQL endpoint and a faceted text search interface. The consistency of the knowledge base was checked with using the OWL2 RL reasoning capabilities of BigOWLIM.

## Results and discussion

Translational medicine requires the full extent of patient data to be accessible so that questions spanning multiple data sources, such as those discussed above, can be asked and answered. For example, a physician in clinical practice would like to easily ask for the criteria for the diagnosis of a disease and the recommendations for personalized medicines. However, TMKB has the potential to be equally relevant to scientists developing new pharmaceutical products. While simple questions may be answered by queries on a single data set, other scientific questions may be addressed only when diverse data sets are fully integrated [38]. Importantly, answering more sophisticated questions may require inference i) over the subclass hierarchy of TMO types or ii) through equivalence mappings. Examples of queries that can now be executed with SPARQL are listed in Table 1, with the full list available on the public wiki.

One ongoing issue in translational informatics is patient privacy and the security of data. An approach that has been pursued using semantic technologies is to encode data access rules and then check all data accesses against these policies [39]. For example, a policy can give a hospital billing specialist access to data about procedures performed at the hospital for the purpose of insurance billing. Then, when procedure data is requested, the requester would need to show that they were a billing specialist and provide the purpose for which they want to access the data. Semantic technologies can be and have been used to encode the policies, recognize compliance (or non-compliance), and explain results.

### SPARQL queries

To demonstrate the utility of the TMO and TMKB, we created fourteen questions to represent the intent of the use case. The questions have been included in this section of the document and are available on the public wiki. The wiki also contains the SPARQL source code and a clickable link that runs the query against the TMKB and displays the results. Fourteen exemplar questions are present on the wiki site with corresponding SPARQL source code and a hyperlink to the results of the first ten. The fourteen queries are reproduced below. The SPARQL source code and results are presented for two selected queries. These queries use the synthetic patient data. To run the queries, click on the link (where provided) or copy the text of the SPARQL query, paste it into the query text box at http://tm.semanticscience.org/sparql and click on “Run Query” button.

The significance of the SPARQL queries we present is to demonstrate that several different types of investigation, spanning information from different disciplines, can be carried out from the same query interface. In the hospital or clinic, the often fragmented information systems do not interoperate, requiring analogous investigations to coordinate between different specialists with access to different types of information. The combination of disparate types of information sources such as EHRs with clinical trial information, information about drugs and adverse reactions, as well as information about genetic variants, is crucial to reaching the goals of personalized medicine. It is precisely this type of information integration that is enabled by linked data approaches such as the one described here.

1. How many patients experienced side effects while taking Donepezil?

2. What are the diagnostic criteria for Alzheimer’s Disease (AD)?

3. Is Donepezil covered by Medicare Part D?

4. Have any of my AD patients been treated for other neurological conditions as this might impact their diagnosis?

5. Are there other clinical trials that my patient may participate in for AD which have a different mechanism of action than the patient’s current drug because it caused side effects?

6. Are there any AD patients without the APOE4 allele as these would be good candidates for the clinical trial involving Bapineuzumab?

7. What active trials are ongoing that would be a good fit for Patient 2?

8. Do I have suitable patients for an AD trial where they are looking for females who are aged over 55 years, have the APOE variant, and low ADAS COG scores?

9. What genes are associated with or implicated in AD?

10. What biomarkers are associated with or implicated in AD?

11. An APOE variant is strongly correlated with AD predisposition. Are there drug classes and drugs that target APOE?

12. Which existing marketed drugs might potentially be re-purposed for AD because they are known to modulate genes that are implicated in the disease?

13. What are the results of patient Georg Steffen Möller’s lipid panel?

14. What is patient Monica Mary Mall’s platelet count over time?

Finding eligible patients can be a costly endeavor for clinical trials so systems that facilitate this activity can save significant costs, as well as increase the effectiveness of treatment. The following query demonstrates the ability to perform patient eligibility studies when the appropriate information is accessible. The use case involves identifying patients without the APOE4 genetic allele for a particular clinical trial. APOE4 is one of three isoforms of Apolipoprotein E in which individuals having one or more copies of the ApoE4 variant exhibit an increased risk of developing late onset (type 2) Alzheimer’s Disease.

Query #6: Are there any AD patients without the APOE4 allele as these would be good candidates for the clinical trial involving Bapineuzumab?

The corresponding SPARQL query is:

The results to this query are listed in table 5.

**Table 5:** Query results for query #6

name	patient
Benny Smith	http://tag:ericw3.org:2009/pchr/3#me
Georg Steffen Möller	http://tag:ericw3.org:2009/pchr/5#me

AD patients in TMKB without the APOE4 allele as these would be good candidates for the clinical trial involving Bapineuzumab.

This next query presents an example of discovering novel uses for existing marketed drugs. We understand this to be of interest to the pharmaceutical industry because of the huge savings in time and money for development and clinical trials. The benefits also translate to physicians and patients because medicines may be available sooner to help manage medical conditions. This query takes advantage of the information in PharmGKB, in which the relations between genes, drugs, and diseases are tracked.

Qquery #12: Which existing marketed drugs might potentially be candidates for AD because they are known to modulate genes that are implicated in the disease?

The corresponding SPARQL query is:

The first 25 results to this query are listed in table 6.

**Table 6:** The first 25 query results for query #12

drug name	disease2 _name
(s)-rolipram	Schizophrenia
(s)-rolipram	Autistic Disorder
(s)-rolipram	Bipolar Disorder
(s)-rolipram	Depression
ACE INHIBITORS, PLAIN	Angioneurotic Edema
ACE INHIBITORS, PLAIN	Hypertension
ACE INHIBITORS, PLAIN	Hypertrophy, Left Ventricular
ACE INHIBITORS, PLAIN	Coronary Disease
ACE INHIBITORS, PLAIN	Alzheimer Disease
ACE INHIBITORS, PLAIN	nondiabetic proteinuric nephropathy
ACE INHIBITORS, PLAIN	Alcoholism
ACE INHIBITORS, PLAIN	Abnormalities
ACE INHIBITORS, PLAIN	Fetal Death
ACE INHIBITORS, PLAIN	Cardiovascular Abnormalities
ACE INHIBITORS, PLAIN	Cardiovascular Diseases
ACE INHIBITORS, PLAIN	Cough
ACE INHIBITORS, PLAIN	Heart Failure
ACE INHIBITORS, PLAIN	Kidney Diseases
ANGIOTENSIN II ANTAGONISTS AND CALCIUM CHANNEL BLOCKERS	Cardiovascular Diseases
ANGIOTENSIN II ANTAGONISTS AND CALCIUM CHANNEL BLOCKERS	Hypertension
ANTIPSYCHOTICS	Schizophrenia
BETA BLOCKING AGENTS	Abnormalities
BETA BLOCKING AGENTS	Fetal Death
BETA BLOCKING AGENTS	Cardiovascular Abnormalities
atenolol	glomerulosclerosis
...	...

A selection of existing marketed drugs in TMKB that might potentially be candidates for AD because they are known to modulate genes that are implicated in the disease.

### Related work

Translational medicine, the integration of the research pipeline from bench to bedside and back, has been a high priority for national biomedical research programs around the world. NIH’s Clinical and Translational Science Awards (CTSAs), set forth by Zerhouni [40], provide leadership in translational research and have been fruitful in producing semantic translational informatics projects [41]. In Europe, Kamel *et al.*[42] introduced the Innovative Medicine Initiative (IMI), a joint undertaking between the European Union and the pharmaceutical industry association, European Federation of Pharmaceutical Industries and Associations (EFPIA). Translational informatics has long been a use case for biomedical semantics. Earlier work by the HCLSIG showed the potential of Semantic Web technologies for translational research [43]. Use cases such as those described in Kashyap *et al.*[44] are being addressed through a number of projects, such as the BRIDG model, a joint project between the Clinical Data Interchange Standards Consortium (CDISC), the HL7 Regulated Clinical Research Information Management Technical Committee (RCRIM TC), the National Cancer Institute (NCI), and the US Food and Drug Administration (FDA). The goal is to produce a shared view of the dynamic and static semantics for protocol-driven research. [45] Other efforts have included development of large-scale terminologies, such as the NCI Thesaurus [46] and the Systematized NOmenclature of MEDicine Clinical Terms (SNOMED CT) [47]. The Informatics for Integrating Biology and the Bedside (i2b2) [48] project has developed a platform to integrate data from diverse sources, including free text and structured databases.

## Conclusions

The Translational Medicine Ontology supports translational medicine by providing a model that facilitates interoperability of data from bench to bedside. Our Alzheimer’s Disease focused use case demonstrates the use of the Translational Medicine Knowledge Base in translational research in the context of a well known disease. The TMKB has also been shown as a good candidate for providing more personalized information for patient treatment. While the medical history of our sample patients is not extensive, it reflects the reality of incomplete medical records in practice today within many institutions. Consistency and completeness of Electronic Health Records will be increasingly important in collaborations between researchers and physicians. More effective integration of data, as we have demonstrated here through the use of applied ontological methods, should enable data mining in a clinical setting to identify superior efficacy of certain drugs over others in specific sections of the population. “Patterns” detected in large data repositories can only be accurately detected if the form and consistency of data is assured. “Noisy” or contaminated data can generate false patterns or generate sufficient noise that true patterns are undetected. A clinician should be able to efficiently obtain a list of safe, effective, evidence-based therapies for administration to a specific patient while considering what payers can afford.

Since our work specifically focused on integrating existing datasets using a common vocabulary, we inevitably acquired terms that are either difficult to define within the context of the TMO or cannot be found in an existing community ontology. For example, the term “side effect” is particularly challenging because side effects in themselves are so varied in their classifications. For example, nightmares are considered processes, but tender gums are dispositions that are realized in processes (sensation of pain in gums when palpated). While the TMO has “adverse drug event” (TMO 0043), it will take time and effort to correctly assign the full set of side effects listed in SIDER.

In addition to the significant health related need for a uniform ontology, in the US, there are now approximately 55 Clinical and Translational Science Centers with approximately 5 more centers to be funded. Each center provides a robust informatics core supporting the entire spectrum of translational science activity. At present, approximately half of the funded centers and some additional 20 research and commercial biomedical research groups around the world use Harvard Medical School’s i2b2 platform. The i2b2 system provides a tremendous opportunity to test TMO’s impact in a broad collection of translational medicine programs and projects. We intend to incorporate the current release of TMO into the i2b2 platform and design a set of pilot projects using TMO to accelerate the research and clinical efforts.

Future work will focus on entities related to drug discovery and drug development in order to increase its utility for the pharmaceutical industry. We aim to incorporate pathway references [49] to support a greater number of pharmaceutical industry use cases. A broader goal is to enable interoperability with large scale e-Science work [50][51]. In order to do this, the underlying representation needs to be expanded to include provenance. Encodings could be done in a provenance interlingua such as the Proof Markup Language [52] or the Open Provenance Model [53]. Sahoo has proposed a method for recording provenance information directly in RDF [54]. Many interdisciplinary e-Science efforts find that they need to provide services to access information, such as the sources relied on to generate a conclusion, the transformations applied to the data, or assumptions embodied in the data. Further, we hope to support deeper semantic scientific knowledge integration [55]. We also hope to engage in the evaluation of data to identify potential inconsistencies and readiness for use. We have utilized logical consistency checking, such as the services available by state of the art OWL reasoners, but we may expand to either utilize or build evaluation services that may, for example, check instance data for possible problems, such as those encountered at the border between open and close-world reasoning [56]. Given the project’s reliance on equivalence links, we may explore using other types of equivalence or similarity relationships, such as those in [57], [58].

Another key goal is the development of a role-based user interface that would encourage vendors of EHRs to use ontologies, such as the TMO, and ontology-enhanced services not only to guide question answering, but also to improve representation and integration of data [59]. The TMKB is intended to provide a first step towards normalizing the sharing and integration of research and clinical artifacts. We wish to enable scientists to capitalize on the benefits derived from open data, communities of practice, and Semantic Web technology for reasoning across vast amounts of health care and life science data. The TMO can also be used to power a set of ontology-enhanced services, such as ontology-enhanced search, provenance, and verification services, thus helping to improve accuracy, trust, and accountability of scientific information. And lastly, we would like to support emerging semantic publishing, referencing, and authoring efforts such as SPAR [60] or SALT [61] by including references to terms in those ontologies.

## Authors' contributions

BA contributed to discussions and provided pharma perspectives on use cases.

CB was involved in the original development of the OWL2 ontology and contributed to the formal ontological structure of TMO and the discussion of the ontological challenges of side effects.

TC reviewed the manuscript and provided information on the AD use case.

CD is a clinician and helped to develop each of the exemplar patient records while providing clinical guidance and support.

CKD participated in TMO use case development, developed roles and interests, contributed to the writing and editing of the manuscript, and created sample patient records.

AJ did the work using LinQuer for mapping the data sources.

JK participated in telecons and contributed to the definition of terms.

PK participated in TMO use case development and transformed narrative descriptions into medical coding; created XML structure for family history, immunizations, lifestyle, and encounters with medical coding designations; contributed to draft and final manuscripts.

JSL is a computational and life scientist with expertise in Semantic Web applications; she contributed to the development and mapping of the TMO classes, the LODD mapping, bringing in expertise, and providing guidance in the writing, editing, and scoping of the manuscript.

TL revised the mapping from Indivo to TMO and the SPARQL queries for the TMKB instance data.

MD is the current lead for the HCLSIG Translational Medicine task force; he provided guidance and expertise, implemented the ontology, mappings, data (PharmGKB), SPARQL queries, and contributed to the writing and editing of the manuscript.

MSM co-chaired the HCLSIG, participated in teleconferences, and contributed to the writing and editing of the manuscript.

JPM is a bioinformaticist and computer scientist, and wrote the related work section. He also provided proofreading, overall review, formatting, and coordination between authors.

DLM is an expert in Semantic Web languages and environments and wrote a future work segment, consulted on the semantic approach, and reviewed and edited the paper and its semantic approach.

EP contributed to the development of TMO, to the mapping of TMO classes to other reference ontologies and source vocabularies, and to the writing and editing of the manuscript. ericP and TG expressed patient data in Indivo, created the query testing framework and produced XSLT to map Indivo to TMO.

RLP was involved in discussions of TMO.

MS gave advice during ontology development and worked on knowledge base consistency checks.

SS co-chaired the HCLSIG, coordinated the task force prior to MD, created the patient records and contributed to the development of the TMO.

PJT participated in TMO use case development; confirmed use case consistency and accuracy; contributed to draft and final manuscript.

PLW contributed to the development of TMO, participated in teleconferences, and reviewed the paper. Other authors participated in conference calls or made other noteworthy contributions to the use cases, TMO or TMKB development effort.

## Competing interests

The authors declare that they have no competing interests.

## Supplementary Material

Additional file 1 **Supplement 01 (v03) to “The Translational Medicine Ontology and Knowledge Base: Driving personalized medicine by bridging the gap between bench and bedside”** A supplemental document containing the TMKB SPARQL queries and results created for this manuscript. Click here for file

## References

[B1] Trusheim M Berndt E Douglas F Stratified medicine: strategic and economic implications of combining drugs and clinical biomarkers Nature Reviews Drug Discovery 2007 6 4 287 293 10.1038/nrd2251 17380152

[B2] Woolf S The meaning of translational research and why it matters JAMA 2008 299 2 211 10.1001/jama.2007.26 18182604

[B3] Rodwin M The case for public ownership of patient data JAMA 2009 302 86 10.1001/jama.2009.965 19567445

[B4] Roses A Pharmacogenetics in drug discovery and development: a translational perspective Nature Reviews Drug Discovery 2008 7 10 807 817 10.1038/nrd2593 18806753

[B5] Centers for Medicare & Medicaid Services (CMS) Medicare & Medicaid EHR Incentive Program Meaningful Use Web Site http://www.cms.gov/EHRIncentivePrograms/35_Meaningful_Use.asp

[B6] Office of the National Coordinator for Health Information Technology (ONCHIT) Standards & Certification Criteria Web Site http://healthit.hhs.gov/portal/server.pt/community/healthit/hhs/gov/standards/ifr/1195

[B7] Luciano JS Negishi M Cohen MA Samson JA Reggia J, Ruppin E, Berndt R Depression Research: Modeling to Illuminate Darkness Neural Modeling of Cognitive and Brain Disorders 1996 World Scientific Publishing Company

[B8] Luciano JS Neural Network Modeling of Unipolar Depression: Patterns of Recovery and Prediction of Outcome PhD thesis 1996 Boston University

[B9] Levine M Calvanio R The Recording of Personal Information as an Intervention and as an Electronic Health Support 2007 Springer

[B10] Calvanio R Buonanno F Levine D Levine M Neuropsychiatric sequelae and life events: Analysis and management6th World Stroke Congress 2008

[B11] Health Care and Life Sciences Pathology Radiology Correlation http://esw.w3.org/HCLSIG/Terminology/PathRadCorrelation

[B12] Allemang D Hendler J Semantic Web for the Working Ontologist: Effective Modeling in RDFS and OWL 2008 Morgan Kaufmann

[B13] Deus H Zhao J Sahoo S Samwald M Prud’hommeaux E Miller M Marshall M Cheung KH Provenance of Microarray Experiments for a Better Understanding of Experiment Results Proceedings of The Second International Workshop on the role of Semantic Web in Provenance Management 2010 Shanghai, China

[B14] McGuinness D Pinheiro da Silva P Explaining answers from the semantic web: The inference web approach Web Semantics: Science, Services and Agents on the World Wide Web 2004 1 4 397 413 10.1016/j.websem.2004.06.002

[B15] Dumontier M Villanueva-Rosales N Towards pharmacogenomics knowledge discovery with the semantic web Briefings in Bioinformatics 2009 10 2 153 10.1093/bib/bbn056 19240125

[B16] Coulet A Smail-Tabbone M Napoli A Devignes M Suggested Ontology For Pharmacogenomics (SO-Pharm): Modular Construction And Preliminary Testing Lecture Notes in Computer Science 2006 4277 648 657 full_text

[B17] Arikuma T Yoshikawa S Azuma R Watanabe K Matsumura K Konagaya A Drug interaction prediction using ontology-driven hypothetical assertion framework for pathway generation followed by numerical simulation BMC Bioinformatics 2008 9 Suppl 6 S11 10.1186/1471-2105-9-S6-S11 18541046 PMC2423434

[B18] Luciano JS PAX of mind for pathway researchers Drug Discovery Today 2005 10 13 937 942 10.1016/S1359-6446(05)03501-4 15993813

[B19] Demir E Cary MP Paley S Fukuda K Lemer C Vastrik I Wu G D’Eustachio P Schaefer C Luciano J Schacherer F Martinez-Flores I Hu Z Jimenez-Jacinto V Joshi-Tope G Kandasamy K Lopez-Fuentes AC Mi H Pichler E Rodchenkov I Splendiani A Tkachev S Zucker J Gopinath G Rajasimha H Ramakrishnan R Shah I Syed M Anwar N Babur O Blinov M Brauner E Corwin D Donaldson S Gibbons F Goldberg R Hornbeck P Luna A Murray-Rust P Neumann E Reubenacker O Samwald M van Iersel M Wimalaratne S Allen K Braun B Whirl-Carrillo M Cheung KHH Dahlquist K Finney A Gillespie M Glass E Gong L Haw R Honig M Hubaut O Kane D Krupa S Kutmon M Leonard J Marks D Merberg D Petri V Pico A Ravenscroft D Ren L Shah N Sunshine M Tang R Whaley R Letovksy S Buetow KH Rzhetsky A Schachter V Sobral BS Dogrusoz U McWeeney S Aladjem M Birney E Collado-Vides J Goto S Hucka M Le Novère N Maltsev N Pandey A Thomas P Wingender E Karp PD Sander C Bader GD The BioPAX community standard for pathway data sharing Nature biotechnology 2010 28 9 935 942 10.1038/nbt.1666 20829833 PMC3001121

[B20] Shah N Jonquet C Chiang A Butte A Chen R Musen M Ontology-driven indexing of public datasets for translational bioinformatics BMC Bioinformatics 2009 10 Suppl 2 S1 10.1186/1471-2105-10-S2-S1 19208184 PMC2646250

[B21] Jentzsch A Zhao J Hassanzadeh O Cheung K Samwald M Andersson B Linking open drug data Triplification Challenge of the International Conference on Semantic Systems 2009 Citeseer

[B22] Patterson C Feightner J Garcia A Hsiung G MacKnight C Sadovnick A Diagnosis and treatment of dementia: 1. Risk assessment and primary prevention of Alzheimer disease Canadian Medical Association Journal 2008 178 5 548 10.1503/cmaj.070796 18299540 PMC2244657

[B23] Minati L Edginton T Grazia Bruzzone M Giaccone G Reviews: Current Concepts in Alzheimer’s Disease: A Multidisciplinary Review American Journal of Alzheimer’s Disease and Other Dementias 2009 24 2 95 10.1177/1533317508328602 PMC10846154 19116299

[B24] Jack C Wiste H Vemuri P Weigand S Senjem M Zeng G Bernstein M Gunter J Pankratz V Aisen P Brain beta-amyloid measures and magnetic resonance imaging atrophy both predict time-to-progression from mild cognitive impairment to Alzheimer’s disease Brain 2010 133 11 3336 10.1093/brain/awq277 20935035 PMC2965425

[B25] Kolate G Sharing of Data Leads to Progress on Alzheimer’s New York Times 2010 Http://www.nytimes.com/2010/08/13/health/research/13alzheimer.html

[B26] HCLS Translational Medicine Task Force http://www.w3.org/wiki/HCLSIG/PharmaOntology

[B27] Scheuermann R Ceusters W Smith B Toward an ontological treatment of disease and diagnosis Proceedings of the 2009 AMIA Summit on Translational Bioinformatics 2009 116 120 PMC3041577 21347182

[B28] Smith B Ceusters W Klagges B Kohler J Kumar A Lomax J Mungall C Neuhaus F Rector A Rosse C Relations in biomedical ontologies Genome Biology 2005 6 5 R46 10.1186/gb-2005-6-5-r46 15892874 PMC1175958

[B29] Noy N Shah N Whetzel P Dai B Dorf M Griffith N Jonquet C Rubin D Storey M Chute C Musen M BioPortal: ontologies and integrated data resources at the click of a mouse Nucleic Acids Res 2009 1 37 10.1093/nar/gkp440 PMC2703982 19483092

[B30] UMLS http://www.nlm.nih.gov/research/umls/

[B31] Dubois B Feldman H Jacova C DeKosky S Barberger-Gateau P Cummings J Delacourte A Galasko D Gauthier S Jicha G Research criteria for the diagnosis of Alzheimer’s disease: revising the NINCDS-ADRDA criteria The Lancet Neurology 2007 6 8 734 746 10.1016/S1474-4422(07)70178-3 17616482

[B32] Knox C Law V Jewison T Liu P Ly S Frolkis A Pon A Banco K Mak C Neveu V Djoumbou Y Eisner R Guo A Wishart D DrugBank 3.0: a comprehensive resource for ’omics’ research on drugs Nucleic Acids Res 2011 Database Issue D1035 41 10.1093/nar/gkq1126 21059682 PMC3013709

[B33] Thorn C Klein T Altman R Pharmacogenomics and bioinformatics: PharmGKB Pharmacogenomics 2010 4 501 5 10.2217/pgs.10.15 PMC3098752 20350130

[B34] Belleau F Nolin M Tourigny N Rigault P Morissette J Bio2RDF: Towards a mashup to build bioinformatics knowledge systems Journal of Biomedical Informatics 2008 41 5 706 716 10.1016/j.jbi.2008.03.004 18472304

[B35] The Indivo Personally Controlled Health Record http://indivohealth.org

[B36] Hassanzadeh O Kementsietsidis A Lim L Miller R Wang M A framework for semantic link discovery over relational data Proceedings of the 18th ACM Conference on Information and Knowledge Management 2009 ACM 1027 1036 full_text

[B37] Volz J Bizer C Gaedke M Kobilarov G Silk–a link discovery framework for the web of data Proceedings of the 2nd Linked Data on the Web Workshop 2009

[B38] Stephens S LaVigna D DiLascio M Luciano J Aggregation of bioinformatics data using Semantic Web technology Web Semant 2006 4 216 221 http://portal.acm.org/citation.cfm?id=1222219.1222307

[B39] Weitzner D Abelson H Berners-Lee T Hanson C Hendler J Kagal L McGuinness D Sussman G Waterman K Transparent accountable inferencing for privacy risk management AAAI Spring Symposium on The Semantic Web meets eGovernment 2006 AAAI Press, Stanford University, USA, Citeseer

[B40] Zerhouni E Translational and clinical science–time for a new vision New England Journal of Medicine 2005 353 15 1621 10.1056/NEJMsb053723 16221788

[B41] Mirhaji P Zhu M Vagnoni M Bernstam E Zhang J Smith J Ontology driven integration platform for clinical and translational research BMC bioinformatics 2009 10 Suppl 2 S2 10.1186/1471-2105-10-S2-S2 19208190 PMC2646248

[B42] Kamel N Compton C Middelveld R Higenbottam T Dahlén S The Innovative Medicines Initiative (IMI): a new opportunity for scientific collaboration between academia and industry at the European level European Respiratory Journal 2008 31 5 924 10.1183/09031936.00033208 18448501

[B43] Ruttenberg A Clark T Bug W Samwald M Bodenreider O Chen H Doherty D Forsberg K Gao Y Kashyap V Kinoshita J Luciano J Marshall M Ogbuji C Rees J Stephens S Wong G Wu E Zaccagnini D Hongsermeier T Neumann E Herman I Cheung K Advancing translational research with the Semantic Web BMC Bioinformatics 2007 8 Suppl 3 S2 10.1186/1471-2105-8-S3-S2 17493285 PMC1892099

[B44] Kashyap V Hongsermeier T Can semantic web technologies enable translational medicine? Semantic Web 2007 249 279 full_text

[B45] Biomedical Research Integrated Domain Group http://www.bridgmodel.org

[B46] Sioutos N Coronado S Haber M Hartel F Shaiu W Wright L NCI Thesaurus: a semantic model integrating cancer-related clinical and molecular information Journal of Biomedical Informatics 2007 40 30 43 10.1016/j.jbi.2006.02.013 16697710

[B47] Stearns M Price C Spackman K Wang A SNOMED clinical terms: overview of the development process and project status Proceedings of the AMIA Symposium 2001 American Medical Informatics Association 662 PMC2243297 11825268

[B48] Murphy S Weber G Mendis M Gainer V Chueh H Churchill S Kohane I Serving the enterprise and beyond with informatics for integrating biology and the bedside (i2b2) Journal of the American Medical Informatics Association 2010 17 2 124 10.1136/jamia.2009.000893 20190053 PMC3000779

[B49] Luciano J Stevens R e-Science and biological pathway semantics BMC Bioinformatics 2007 8 Suppl 3 S3 10.1186/1471-2105-8-S3-S3 17493286 PMC1892100

[B50] Hey T Trefethen A Cyberinfrastructure for e-Science Science 2005 308 5723 817 821 10.1126/science.1110410 15879209

[B51] Hey T Trefethen A e-Science and its implications Philos Transact A Math Phys Eng Sci 2003 361 1809 1809 1825 10.1098/rsta.2003.1224 12952686

[B52] McGuinness D Ding L da Silva P Chang C Pml 2: A modular explanation interlingua Proceedings of AAAI 2007 7

[B53] Moreau L Clifford B Freire J Futrelle J Gil Y Groth P Kwasnikowska N Miles S Missier P Myers J The open provenance model core specification (v1. 1) Future Generation Computer Systems 2010

[B54] Sahoo S Bodenreider O Hitzler P Sheth A Thirunarayan K Provenance Context Entity (PaCE): Scalable provenance tracking for scientific RDF data Proceedings of the 22nd International Scientific and Statistical Database Management (SSDBM) Conference 2010 SSDBM 461 470 10.1007/978-3-642-13818-8_32 PMC4303908 25621321

[B55] McGuinness D Fox P Brodaric B Kendall E The Emerging Field of Semantic Scientific Knowledge Integration IEEE Intelligent Systems 2009 25 26

[B56] Tao J Ding L McGuinness D Instance data evaluation for semantic web-based knowledge management systems System Sciences, 2009. HICSS'09. 42nd Hawaii International Conference on 2009 IEEE 1 10

[B57] Halpin H Hayes P McCusker J McGuinness D Thompson H When owl: sameAs isn’t the Same: An Analysis of Identity in Linked Data Proc. 9th Int. Semantic Web Conf 2010

[B58] Ding L Shinavier J Shangguan Z McGuinness D SameAs Networks and Beyond: Analyzing Deployment Status and Implications of owl: sameAs in Linked Data Proc. 9th Int. Semantic Web Conf 2010

[B59] Goble C Pettifer S Stevens R Greenhalgh C Knowledge Integration: In Silico Experiments in Bioinformatics The Grid: Blueprint for a New Computing Infrastructure 2003 121 134

[B60] Semantic Publishing and Referencing http://esw.w3.org/HCLSIG/SWANSIOC/Actions/RhetoricalStructure/meetings/20101115

[B61] Semantically Annotated LaTeX for scientific publications http://www.springerlink.com/content/t220214924577133/

