# Supplementary material for: The Translational Medicine Ontology and Knowledge Base: driving personalized medicine by bridging the gap between bench and bedside
Source: J Biomed Semantics. 2011 May 17;2(Suppl 2):S1. doi: 10.1186/2041-1480-2-S2-S1 (PMC3102889; doi:10.1186/2041-1480-2-S2-S1)
Supplement: Additional file 1 — Supplement 01 (v03) to “The Translational Medicine Ontology and Knowledge Base: Driving personalized medicine by bridging the gap between bench and bedside” A supplemental document containing the TMKB SPARQL queries and results created for this manuscript. [file 2041-1480-2-S2-S1-S1.pdf]

File contents:

Supplement 01 (v03) to "The Translational Medicine Ontology and Knowledge Base: Driving personalized medicine by bridging the gap between bench and bedside", Joanne S. Luciano et al., JBMS, Bio-Ontologies 2010 (2011)

Comments:

This supplement contains sample SPARQL queries relevant for the paper listed above. All of the queries are functional as of 7-Feb-2011 and return the listed results. Depending on future changes to TMO and the TMOKB, queries and results might change in the future. These queries and links to the results are also listed at:

<http://esw.w3.org/HCLSIG/PharmaOntology/Queries>.

TMO use case related queries

Q1. How many patients experienced side effects while taking Donepezil?

SPARQL

PREFIX indivo: <<http://indivo.org/vocab/xml/documents#>>

PREFIX trans: <tag:eric@w3.org:2009/tmo/translator#>

PREFIX foaf: <<http://xmlns.com/foaf/0.1/>>

```
SELECT DISTINCT(?patient) ?patient_name ?encounter_date ?symptoms ?diagnosis
?drug_name ?prescription_start
WHERE {
  GRAPH <pchr> {
    ?patient foaf:name ?patient_name .
    ?encounter trans:patient ?patient ;
      trans:reportedSymptoms ?symptoms ;
      trans:workingDiagnosis ?diagnosis ;
      trans:dateOfEncounter ?encounter_date .
    ?drug trans:prescribedTo ?patient ;
      indivo:prescribedOn ?prescription_start ;
      trans:datePrescriptionEnded ?prescription_end ;
      trans:genericName ?drug_name .
    FILTER regex(?drug_name, "DONEPEZIL", "i") .
    FILTER (?prescription_start <= ?encounter_date && ?prescription_end >=
?encounter_date) .
  }
}
```

Results

patient	patient_name	encounter_date	symptoms	diagnosis	drug_name	prescription_start
http://tag:eric@w3.org:2009/pchr/1#me	George Andrew Tour	2009-08-15	nausea	adverse event to Donepezil	DONEPEZIL HYDROCHLORIDE TABLETS	2009-08-10

Supplement 01 (v03) to "The Translational Medicine Ontology and Knowledge Base: Driving personalized medicine by bridging the gap between bench and bedside", Joanne S. Luciano et al., JBMS, Bio-Ontologies 2010 (2011)

Q2. What are the diagnostic criteria for AD?

SPARQL

define input:inference "infer"

PREFIX tmo: <http://www.w3.org/2001/sw/hcls/ns/transmed/>

PREFIX iao: <http://purl.obolibrary.org/obo/>

PREFIX ro: <http://www.obofoundry.org/ro/ro.owl#>

SELECT DISTINCT ?type_label ?criterion_label

WHERE {

    [] a ?type ;

        a ?stated_type ;

        rdfs:label ?criterion_label ;

        iao:is_about <http://www4.wiwiw.fu-berlin.de/diseasome/resource/diseases/74> .

    ?stated_type rdfs:label ?type_label .

    ?stated_type rdfs:subClassOf ?type .

    FILTER (?type = tmo:TMO_0068) .

}

ORDER BY DESC(?type_label) ASC(?criterion_label)

Results

type_label	criterion_label
diagnostic inclusion criterion	Abnormal cerebrospinal fluid biomarker [dubois:7]
diagnostic inclusion criterion	Both clinical and genetic evidence (mutation on chromosome 1, 14, or 21) of AD [dubois:21]
diagnostic inclusion criterion	Both clinical and histopathological (brain biopsy or autopsy) evidence of the disease, as required by the NIA-Reagan criteria for the post-mortem diagnosis of AD [dubois:20]
diagnostic inclusion criterion	Gradual and progressive change in memory function reported by patients or informants over more than 6 months [dubois:2]
diagnostic inclusion criterion	Low amyloid ?1?42 concentrations, increased total tau concentrations, or increased phospho-tau concentrations, or combinations of the three [dubois:8]
diagnostic inclusion criterion	Objective evidence of significantly impaired episodic memory on testing: this generally consists of recall deficit that does not improve significantly or does not normalise with cueing or recognition testing and after effective encoding of information has been previously controlled [dubois:3]

diagnostic inclusion criterion	Presence of an early and significant episodic memory impairment [dubois:1]
diagnostic inclusion criterion	Presence of medial temporal lobe atrophy [dubois:5]
diagnostic inclusion criterion	Proven AD autosomal dominant mutation within the immediate family [dubois:10]
diagnostic inclusion criterion	Reduced glucose metabolism in bilateral temporal parietal regions with PET [dubois:9]
diagnostic inclusion criterion	The episodic memory impairment can be isolated or associated with other cognitive changes at the onset of AD or as AD advances [dubois:4]
diagnostic inclusion criterion	Volume loss of hippocampi, entorhinal cortex, amygdala evidenced on MRI with qualitative ratings using visual scoring (referenced to well characterised population with age norms) or quantitative volumetry of regions of interest (referenced to well characterised population with age norms) [dubois:6]
diagnostic exclusion criterion	Cerebrovascular disease [dubois:17]
diagnostic exclusion criterion	Early extrapyramidal signs [dubois:13]
diagnostic exclusion criterion	Early occurrence of the following symptoms: gait disturbances, seizures, behavioural changes [dubois:12]
diagnostic exclusion criterion	Focal neurological features including hemiparesis, sensory loss, visual field deficits [dubois:14]
diagnostic exclusion criterion	MRI FLAIR or T2 signal abnormalities in the medial temporal lobe that are consistent with infectious or vascular insults [dubois:19]
diagnostic exclusion criterion	Major depression [dubois:16]
diagnostic exclusion criterion	Non-AD dementia [dubois:15]
diagnostic exclusion criterion	Sudden onset [dubois:11]
diagnostic exclusion criterion	Toxic and metabolic abnormalities [dubois:18]

Q3. Is Donepezil covered by Medicare D?

SPARQL

define input:inference "infer"

PREFIX tmo: <<http://www.w3.org/2001/sw/hcls/ns/transmed/>>

PREFIX medicare: <<http://www4.wiwiwiss.fu-berlin.de/medicare/resource/medicare/>>

SELECT ?label

WHERE {

    GRAPH <medicare> {

        ?medicare_drug a tmo:TMO_0001 .

Supplement 01 (v03) to "The Translational Medicine Ontology and Knowledge Base: Driving personalized medicine by bridging the gap between bench and bedside", Joanne S. Luciano et al., JBMS, Bio-Ontologies 2010 (2011)

```

    ?medicare_drug rdfs:label ?label .
    FILTER regex(?label, "donepezil", "i") .
  }
}

```

Results

label
Aricept (Donepezil Hydrochloride)
Aricept ODT (Donepezil Hydrochloride)

Q4. Have any of my AD patients been treated for other neurological conditions as this might impact their diagnosis?

SPARQL

```

PREFIX indivo: <http://indivo.org/vocab/xml/documents#>
PREFIX trans: <tag:eric@w3.org:2009/tmo/translator#>
PREFIX foaf: <http://xmlns.com/foaf/0.1/>

```

```

SELECT DISTINCT(?patient) ?patient_name
WHERE {
  GRAPH <pchr> {
    ?patient foaf:name ?patient_name .
    [
      trans:patient ?patient ;
      trans:reportedSymptoms ?symptoms ;
    ]
    FILTER regex(?symptoms, "depression", "i") .
    #ICD9 of 331 - alzheimer disease
    ?patient trans:hasCondition [ trans:ICD9 "331" ; ] .
  }
}

```

Results

patient	patient_name
http://tag:eric@w3.org:2009/pchr/2#me	Monica Mary Mall

Q5. Are there other clinical trials that my patient may participate in for AD which have a different mechanism of action to the patient's current drug because it caused side effects?

A5.1. 569 clinical trials which involve AD are known. 181 trials are enrolling by invitation or recruiting patients.

SPARQL

PREFIX linkedct: <http://data.linkedct.org/resource/linkedct/>

PREFIX drugbank: <http://www4.wiwiss.fu-berlin.de/drugbank/resource/drugbank/>

```
SELECT ?trial_status, count(*)
WHERE {
  ?trial ?type linkedct:trials .
  ?trial linkedct:overall_status ?trial_status .
  ?trial linkedct:condition ?condition .
  ?condition linkedct:condition_name ?condition_name .
  FILTER regex(?condition_name, "alzheimer", "i") .
}
```

Results

trial_status	callret-1
Enrolling by invitation	12
Active, not recruiting	72
Withdrawn	1
Completed	263
Suspended	2
Recruiting	169
Terminated	31
Not yet recruiting	19

A5.2. 72 active clinical trials involve AD.

SPARQL

PREFIX linkedct: <http://data.linkedct.org/resource/linkedct/>

PREFIX drugbank: <http://www4.wiwiss.fu-berlin.de/drugbank/resource/drugbank/>

```
SELECT count(*)
WHERE {
  ?trial ?type linkedct:trials .
  ?trial linkedct:overall_status ?trial_status .
  ?trial linkedct:condition ?condition .
  ?condition linkedct:condition_name ?condition_name .
  FILTER regex(?trial_status, "active", "i") .
  FILTER regex(?condition_name, "alzheimer", "i") .
}
```

Results

callret-0
72

A5.3. 2 drugs targeting AD with established mechanism of action are in clinical trials.

Supplement 01 (v03) to "The Translational Medicine Ontology and Knowledge Base: Driving personalized medicine by bridging the gap between bench and bedside", Joanne S. Luciano et al., JBMS, Bio-Ontologies 2010 (2011)

SPARQL

PREFIX linkedct: <http://data.linkedct.org/resource/linkedct/>

PREFIX drugbank: <http://www4.wiwiw.fu-berlin.de/drugbank/resource/drugbank/>

```
SELECT ?name ?moa
WHERE {
  ?trial ?type linkedct:trials .
  ?trial linkedct:overall_status ?trial_status .
  ?trial linkedct:condition ?condition .
  ?condition linkedct:condition_name ?condition_name .
  ?trial linkedct:intervention ?ldrug .
  ?ldrug linkedct:intervention_name ?name .
  ?ddrug owl:sameAs ?ldrug .
  ?ddrug drugbank:mechanismOfAction ?moa .
  FILTER regex(?trial_status, "active", "i") .
  FILTER regex(?condition_name, "alzheimer", "i") .
}
```

Results

name	moa
placebo	Alkylating agents work by three different mechanisms: 1) attachment of alkyl groups to DNA bases, resulting in the DNA being fragmented by repair enzymes in their attempts to replace the alkylated bases, preventing DNA synthesis and RNA transcription from the affected DNA, 2) DNA damage via the formation of cross-links (bonds between atoms in the DNA) which prevents DNA from being separated for synthesis or transcription, and 3) the induction of mispairing of the nucleotides leading to mutations.
rivastigmine	Rivastigmine increases the concentration of acetylcholine through reversible inhibition of its hydrolysis by cholinesterase. This acts to enhance cholinergic function.

Q6. Are there any AD patients without the APOE4 allele as these would be good candidates for the clinical trial involving Bapineuzumab?

SPARQL

PREFIX trans: <tag:eric@w3.org:2009/tmo/translator#>

PREFIX foaf: <http://xmlns.com/foaf/0.1/>

Supplement 01 (v03) to "The Translational Medicine Ontology and Knowledge Base: Driving personalized medicine by bridging the gap between bench and bedside", Joanne S. Luciano et al., JBMS, Bio-Ontologies 2010 (2011)

```

SELECT DISTINCT ?name ?patient
WHERE {
  ?patient trans:hasCondition [ trans:diagnosedWith trans:alzheimers_disease ] ;
    foaf:name ?name .
  OPTIONAL {
    ?encounter trans:test [
      a ?testname ;
      trans:result ?result
    ] .
    ?result trans:feature trans:variant_APOE4 ;
      trans:present true .
  }
  FILTER ( !bound(?result) ) .
}

```

Results

name	patient
Benny Smith	http://tag:eric@w3.org:2009/pchr/3#me
Georg Steffen Möller	http://tag:eric@w3.org:2009/pchr/5#me

Q7. What active trials are ongoing that would be a good fit for Patient 2?

SPARQL

PREFIX linkedct: <http://data.linkedct.org/resource/linkedct/>
 PREFIX trans: <tag:eric@w3.org:2009/tmo/translator#>

```

SELECT DISTINCT(?trial) ?trial_condition_name ?trial_title
WHERE {
  ?trial linkedct:brief_title ?trial_title .
  ?trial linkedct:overall_status ?status .
  FILTER regex(?status, "Active") .
  ?trial linkedct:condition ?trial_condition .
  ?trial_condition linkedct:condition_name ?trial_condition_name .
  FILTER (
    regex(?trial_condition_name, "alzheimer", "i")
    || regex(?trial_condition_name, "anxiety", "i")
    || regex(?trial_condition_name, "hypercholesterolaemia", "i")
    || regex(?trial_condition_name, "depression", "i")
    || regex(?trial_condition_name, "mild cognitive impairment", "i")
    || regex(?trial_condition_name, "myocardial infarction", "i")
  ) .
}

```

Results

Supplement 01 (v03) to "The Translational Medicine Ontology and Knowledge Base: Driving personalized medicine by bridging the gap between bench and bedside", Joanne S. Luciano et al., JBMS, Bio-Ontologies 2010 (2011)

trial	trial_condition_name	trial_title
http://data.linkedct.org/resource/trials/NCT00263393	* Myocardial Infarction	Rural Andhra Pradesh Cardiovascular Prevention Study (RAPCAPS)
http://data.linkedct.org/resource/trials/NCT00199823	Acute Anterior Wall Myocardial Infarction	Autologous Stem Cell Transplantation in Acute Myocardial Infarction
http://data.linkedct.org/resource/trials/NCT00211172	Acute Myocardial Infarction	HMO Research Network CERT: Acute Myocardial Infarction
http://data.linkedct.org/resource/trials/NCT00145119	Acute Myocardial Infarction	CARISMA : Cardiac Arrhythmias and Risk Stratification After MyoCardial Infarction
http://data.linkedct.org/resource/trials/NCT00157768	Acute Myocardial Infarction	IRIS : Use of Implantable Defibrillator in High-Risk Patients Early After Acute Myocardial Infarction
http://data.linkedct.org/resource/trials/NCT00161005	Acute Myocardial Infarction	Norwegian Study on District Treatment of ST-Elevation Myocardial Infarction
http://data.linkedct.org/resource/trials/NCT00419198	Acute Myocardial Infarction	Clinical Outcomes of Angioplasty Postconditioning
http://data.linkedct.org/resource/trials/NCT00430885	Acute Myocardial Infarction	Effect of Intravenous Immunglobulin (IVIG) After Myocardial Infarction
http://data.linkedct.org/resource/trials/NCT00623272	Acute Myocardial Infarction	Left Ventricular Function Assessment After Acute Myocardial Infarction: Comparison Between Bi-, Three-Dimensional and Cardiac Magnetic Resonance
http://data.linkedct.org/resource/trials/NCT00669227	Acute Myocardial Infarction	Intracoronary Stem Cell Therapy in Patients With Acute Myocardial Infarction (SCAMI)
http://data.linkedct.org/resource/trials/NCT00409604	Acute Myocardial Infarction	PROTECT : Pacing to Protect Heart for Damage From Blocked Heart Vessel and From re-Opening Blocked Vessel(s)
http://data.linkedct.org/resource/trials/NCT00495664	Acute Myocardial Infarction	Titanium Nitride Oxide Coated Stents and Paclitaxel Eluting Stents for Acute Myocardial Infarction
http://data.linkedct.org/resource	Acute Myocardial Infarction	Counterpulsation Reduces

Supplement 01 (v03) to "The Translational Medicine Ontology and Knowledge Base: Driving personalized medicine by bridging the gap between bench and bedside", Joanne S. Luciano et al., JBMS, Bio-Ontologies 2010 (2011)

/trials/NCT00833612	(AMI)	Infarct Size Pre-PCI for AMI
http://data.linkedct.org/resource/trials/NCT00399776	Adolescent Depression	Bone Mineral Content and Bone Metabolism in Adolescents on Antipsychotic Therapy
http://data.linkedct.org/resource/trials/NCT00009191	Alzheimer Disease	The Depression in Alzheimer's Disease Study (DIADS)
http://data.linkedct.org/resource/trials/NCT00094913	Alzheimer Disease	PET Changes in Alzheimer's Disease (AD)
http://data.linkedct.org/resource/trials/NCT00119561	Alzheimer Disease	Testing the Effectiveness of Telephone Support for Dementia Caregivers
http://data.linkedct.org/resource/trials/NCT00040378	Alzheimer Disease	Prevention of Alzheimer's Disease by Vitamin E and Selenium (PREADVISE)
http://data.linkedct.org/resource/trials/NCT00056329	Alzheimer Disease	Vitamin E in Aging Persons With Down Syndrome
http://data.linkedct.org/resource/trials/NCT00071721	Alzheimer Disease	Valproate in Dementia (VALID)
http://data.linkedct.org/resource/trials/NCT00088673	Alzheimer Disease	Evaluation of 3APS in Patients With Mild to Moderate Alzheimer's Disease
http://data.linkedct.org/resource/trials/NCT00291161	Alzheimer Disease	Partners in Dementia Care
http://data.linkedct.org/resource/trials/NCT00441987	Alzheimer Disease	Study Evaluating the Safety and Pharmacokinetics of a Single Dose of GSI-953
http://data.linkedct.org/resource/trials/NCT00506415	Alzheimer Disease	Comparative Efficacy, Safety, and Tolerability of Rivastigmine 10 and 15 cm2 Patch in Patients With Alzheimer's Disease (AD) Showing Cognitive Decline
http://data.linkedct.org/resource/trials/NCT00547560	Alzheimer Disease	Study Evaluating Multiple Doses of GSI-953 Within the Elderly Population
http://data.linkedct.org/resource/trials/NCT00568776	Alzheimer Disease	ELND005 in Patients With Mild to Moderate Alzheimer's Disease
http://data.linkedct.org/resource/trials/NCT00605059	Alzheimer Disease	Evaluation of [123I] AV94 and SPECT as a Marker of Beta-Amyloid Protein Deposition in Subjects With Alzheimer Disease in Comparison to Healthy

Supplement 01 (v03) to "The Translational Medicine Ontology and Knowledge Base: Driving personalized medicine by bridging the gap between bench and bedside", Joanne S. Luciano et al., JBMS, Bio-Ontologies 2010 (2011)

		Subjects
http://data.linkedct.org/resource/trials/NCT00470418	Alzheimer Disease	Development of NIC5-15 in the Treatment of Alzheimer's Disease
http://data.linkedct.org/resource/trials/NCT00599469	Alzheimer Disease	Far Infrared Treatment for Alzheimer's Disease
http://data.linkedct.org/resource/trials/NCT00733863	Alzheimer Disease	Safety and Tolerability of Repeated Subcutaneous Injections of CAD106 in Mild Alzheimer's Patients.
http://data.linkedct.org/resource/trials/NCT00462917	Alzheimer Disease	REVEAL III: Risk Evaluation and Education for Alzheimer's Disease
http://data.linkedct.org/resource/trials/NCT00605046	Alzheimer Disease	Evaluation of [123I] AV151 and SPECT as a Marker of Beta-Amyloid Protein Deposition in Subjects With Alzheimer Disease in Comparison to Healthy Subjects
http://data.linkedct.org/resource/trials/NCT00824967	Alzheimer Disease	Non Pharmacological Management of Behavioural Symptoms in Nursing Home
http://data.linkedct.org/resource/trials/NCT00979316	Alzheimer Disease	Effect on the Electrocardiographic QT Interval Corrected for Heart Rate (QTc) in Healthy Subjects
http://data.linkedct.org/resource/trials/NCT00627848	Alzheimer' Disease	Rivastigmine in Mild Alzheimer's Disease, FMRI Study
http://data.linkedct.org/resource/trials/NCT00031018	Alzheimer's Disease	Prevention of Cognitive Decline in Alzheimer's Disease by Ingested Interferon Alpha
http://data.linkedct.org/resource/trials/NCT00087789	Alzheimer's Disease	CERE-110 in Subjects With Mild to Moderate Alzheimer's Disease
http://data.linkedct.org/resource/trials/NCT00348140	Alzheimer's Disease	Rosiglitazone (Extended Release Tablets) As Adjunctive Therapy In Subjects With Mild To Moderate Alzheimer's Disease
http://data.linkedct.org/resource/trials/NCT00086138	Alzheimer's Disease	Depression in Alzheimer's Disease-2
http://data.linkedct.org/resource/trials/NCT00106899	Alzheimer's Disease	ADNI: Alzheimer's Disease Neuroimaging Initiative

Supplement 01 (v03) to "The Translational Medicine Ontology and Knowledge Base: Driving personalized medicine by bridging the gap between bench and bedside", Joanne S. Luciano et al., JBMS, Bio-Ontologies 2010 (2011)

http://data.linkedct.org/resource/trials/NCT00142324	Alzheimer's Disease	CALM-AD
http://data.linkedct.org/resource/trials/NCT00217763	Alzheimer's Disease	European Study of 3APS in Mild to Moderate Alzheimer's Disease Patients
http://data.linkedct.org/resource/trials/NCT00239746	Alzheimer's Disease	BONSAI: Biomarkers of Nonsteroidal Anti-Inflammatories
http://data.linkedct.org/resource/trials/NCT00259480	Alzheimer's Disease	Project ACT: Advancing Caregiving Techniques
http://data.linkedct.org/resource/trials/NCT00267124	Alzheimer's Disease	Brain Aging Project-- Kansas University
http://data.linkedct.org/resource/trials/NCT00299988	Alzheimer's Disease	Phase II Study of Intravenous Immunoglobulin (IVIg) for Alzheimer's Disease
http://data.linkedct.org/resource/trials/NCT00314912	Alzheimer's Disease	Open-Label Extension of the Phase III Study With Tramiprosate (3APS) in Patients With Mild to Moderate Alzheimer's Disease
http://data.linkedct.org/resource/trials/NCT00319891	Alzheimer's Disease	Computer-Based Training for Mild Alzheimer's Disease
http://data.linkedct.org/resource/trials/NCT00010803	Alzheimer's Disease	Ginkgo Biloba Prevention Trial in Older Individuals
http://data.linkedct.org/resource/trials/NCT00174525	Alzheimer's Disease	Safety Study of Passive Immunization for Patients With Mild to Moderate Alzheimer's Disease
http://data.linkedct.org/resource/trials/NCT00259467	Alzheimer's Disease	Project TAP: Tailored Activities Project
http://data.linkedct.org/resource/trials/NCT00348309	Alzheimer's Disease	Rosiglitazone (Extended Release Tablets) As Adjunctive Therapy For Subjects With Mild To Moderate Alzheimer's Disease
http://data.linkedct.org/resource/trials/NCT00377715	Alzheimer's Disease	Double-Blind, Placebo-Controlled Study of Oral Dimebon in Subjects With Mild to Moderate Alzheimer's Disease
http://data.linkedct.org/resource/trials/NCT00561392	Alzheimer's Disease	Clinical Effectiveness of 10 cm2 Rivastigmine Patch in Patients With Alzheimer's Disease

Supplement 01 (v03) to "The Translational Medicine Ontology and Knowledge Base: Driving personalized medicine by bridging the gap between bench and bedside", Joanne S. Luciano et al., JBMS, Bio-Ontologies 2010 (2011)

http://data.linkedct.org/resource/trials/NCT00622713	Alzheimer's Disease	A 24 Week, Multicenter, Open, Evaluation of the Clinical Effectiveness of the Once-Daily 10 cm ² Rivastigmine Patch Formulation in Patients With Probable Alzheimer's Disease (EXTRA)
http://data.linkedct.org/resource/trials/NCT00626613	Alzheimer's Disease	The Relationship Between Risperdal Treatment and Quality of Life in Patients With Alzheimer's Disease and Behavioural and Psychological Symptoms of Dementia (BPSD)
http://data.linkedct.org/resource/trials/NCT00675623	Alzheimer's Disease	A Safety and Efficacy Study of Oral Dimebon in Patients With Mild-To-Moderate Alzheimer's Disease
http://data.linkedct.org/resource/trials/NCT00684944	Alzheimer's Disease	Open Label Study of TRx0014 in Alzheimer's Disease
http://data.linkedct.org/resource/trials/NCT00381238	Alzheimer's Disease	Open-Label Extension Assessing Long-Term Safety Of Rosiglitazone In Subjects With Mild To Moderate Alzheimer's Disease
http://data.linkedct.org/resource/trials/NCT00388544	Alzheimer's Disease	Enhancing Quality of Life for Nursing Home Residents
http://data.linkedct.org/resource/trials/NCT00440050	Alzheimer's Disease	DHA (Docosahexaenoic Acid), an Omega 3 Fatty Acid, in Slowing the Progression of Alzheimer's Disease
http://data.linkedct.org/resource/trials/NCT00480701	Alzheimer's Disease	Evaluation of 123-I Iodobenzovesamicol (IBVM) and SPECT Imaging of Healthy Subjects and Patients With Alzheimer's Disease
http://data.linkedct.org/resource/trials/NCT00546767	Alzheimer's Disease	Home-Based Assessment for Alzheimer Disease Prevention
http://data.linkedct.org/resource/trials/NCT00566501	Alzheimer's Disease	Open-Label Extension Study of 23 mg Donepezil SR in Patients With

		Moderate to Severe Alzheimer's Disease
http://data.linkedct.org/resource/trials/NCT00581867	Alzheimer's Disease	Memory and Insulin in Early Alzheimer's Disease
http://data.linkedct.org/resource/trials/NCT00627029	Alzheimer's Disease	Evaluation of Programs of Coordinated Care and Disease Management
http://data.linkedct.org/resource/trials/NCT00704782	Alzheimer's Disease	Study of Combination Therapy With Dimebon and Donepezil (Aricept) in Patients With Alzheimer's Disease
http://data.linkedct.org/resource/trials/NCT00438568	Alzheimer's Disease	SNIFF 120: Study of Nasal Insulin to Fight Forgetfulness (120 Days)
http://data.linkedct.org/resource/trials/NCT00478205	Alzheimer's Disease	Comparison of 23 mg Donepezil Sustained Release (SR) to 10 mg Donepezil Immediate Release (IR) in Patients With Moderate to Severe Alzheimer's Disease
http://data.linkedct.org/resource/trials/NCT00531609	Alzheimer's Disease	Safety and Tolerability of Long-Term Treatment by Rivastigmine Transdermal Patch in Patients With Probable Alzheimer's Disease
http://data.linkedct.org/resource/trials/NCT00594568	Alzheimer's Disease	Effect of LY450139 on the Long Term Progression of Alzheimer's Disease
http://data.linkedct.org/resource/trials/NCT00606476	Alzheimer's Disease	AAB-001 (Bapineuzumab) Open-Label, Long-Term Extension Study in Patients With Mild to Moderate Alzheimer's Disease
http://data.linkedct.org/resource/trials/NCT00759863	Alzheimer's Disease	LifeZig Personalized Reminiscence Video With Slideshows and Music for Individuals With Alzheimer's and Dementia
http://data.linkedct.org/resource/trials/NCT00777608	Alzheimer's Disease	A Study to Test the Performance of the CogState Computerized Neuropsychological Battery in Patients With Alzheimer's Disease.
http://data.linkedct.org/resource/trials/NCT00889603	Alzheimer's Disease	Non-Interventional Study With Aricept® Evess

Supplement 01 (v03) to "The Translational Medicine Ontology and Knowledge Base: Driving personalized medicine by bridging the gap between bench and bedside", Joanne S. Luciano et al., JBMS, Bio-Ontologies 2010 (2011)

http://data.linkedct.org/resource/trials/NCT00795418	Alzheimer's Disease	Safety and Tolerability of Repeated Subcutaneous Injections of CAD 106 in Mild Alzheimer's Patients
http://data.linkedct.org/resource/trials/NCT00810147	Alzheimer's Disease	A Phase II, Multicenter, Double Blind, Placebo-Controlled Safety, Tolerability Study of BMS-708163 in Patients With Mild to Moderate Alzheimer's Disease
http://data.linkedct.org/resource/trials/NCT00757939	Alzheimer's Disease	A Study to Evaluate dASL Imaging as a Biomarker for Alzheimer's Disease
http://data.linkedct.org/resource/trials/NCT00829816	Alzheimer's Disease	Safety and Tolerability of Dimebon in Patients on Memantine, and Memantine Plus Donepezil
http://data.linkedct.org/resource/trials/NCT00880347	Alzheimer's Disease	Blood Gene Expression Signature in Patients Diagnosed With Probable Alzheimer's Disease Compared to Patients Suffering From Other Types of Dementia
http://data.linkedct.org/resource/trials/NCT00987090	Alzheimer's Disease	Early-onset and Late-onset Sporadic Alzheimer's Disease (AD)
http://data.linkedct.org/resource/trials/NCT00988624	Alzheimer's Disease	A Study In Healthy Volunteers To Estimate The Pharmacokinetics Of Four Modified-Release Formulations Of Dimebon (Latrepirdine)
http://data.linkedct.org/resource/trials/NCT00670228	Anterior Wall Myocardial Infarction	EFFECT of Insulin Glargine and Apidra in Patients With Acute ST Elevation MI
http://data.linkedct.org/resource/trials/NCT00303147	Anxiety	Cognitive and Health Benefits of Expressive Writing for Family Caregivers Under Stress
http://data.linkedct.org/resource/trials/NCT00164203	Anxiety	The Feelings Club: Evaluating a School-Based Intervention for Children at Risk for Depression and Anxiety Disorders
http://data.linkedct.org/resource/trials/NCT00245817	Anxiety	HINCAB-Study. Home Based Intervention in

Supplement 01 (v03) to "The Translational Medicine Ontology and Knowledge Base: Driving personalized medicine by bridging the gap between bench and bedside", Joanne S. Luciano et al., JBMS, Bio-Ontologies 2010 (2011)

		Nursing for Coronary Artery Bypass Patients
http://data.linkedct.org/resource/trials/NCT00108277	Anxiety	Respiratory Dysregulation and Breathing Training in Anxious Outpatients
http://data.linkedct.org/resource/trials/NCT00152763	Anxiety	Psychological Support for Patients With an Implantable Cardioverter Defibrillator
http://data.linkedct.org/resource/trials/NCT00183326	Anxiety	Treating Post Traumatic Stress Disorder in Children Exposed to Domestic Violence
http://data.linkedct.org/resource/trials/NCT00302744	Anxiety	Effects of Psilocybin in Advanced-Stage Cancer Patients With Anxiety
http://data.linkedct.org/resource/trials/NCT00360451	Anxiety	Effectiveness of School-Based Cognitive Behavioral Therapy in Preventing Depression in Young Adolescents
http://data.linkedct.org/resource/trials/NCT00578214	Anxiety	Safety and Efficacy of Oral Midazolam for Perioperative Anxiety Relief of Patients Undergoing Mohs Micrographic Surgery
http://data.linkedct.org/resource/trials/NCT00634608	Anxiety	Integrating Targeted MedlinePlus Health Prescriptions Into Clinic Practice Workflow
http://data.linkedct.org/resource/trials/NCT00641940	Anxiety	Girls in Transition Study: Helping Girls Enter the Teenage Years
http://data.linkedct.org/resource/trials/NCT00841022	Anxiety	Information and Anaesthesia in Paediatrics
http://data.linkedct.org/resource/trials/NCT00114127	Anxiety Disorder	Duloxetine for Social Anxiety Disorder: Prediction of Long Term Outcome
http://data.linkedct.org/resource/trials/NCT00225459	Anxiety Disorder	Does Spiritual/Religious Commitment Mediate the Relationship Between Mood Variability and Suicidal Ideation.
http://data.linkedct.org/resource/trials/NCT00438685	Anxiety Disorder	Outpatient Adolescent Treatment for Comorbid Substance Use and Internalizing Disorders

http://data.linkedct.org/resource/trials/NCT00483639	Anxiety Disorder	Study of Anxiety and Avoidance of Others in Patients Previously Treated for Head and Neck Cancer
http://data.linkedct.org/resource/trials/NCT00373022	Anxiety Disorder	Moderate Physical Activity in Helping Patients Recover Physically and Emotionally From Stage II Or Stage III Colorectal Cancer
http://data.linkedct.org/resource/trials/NCT00158327	Anxiety Disorders	Telephone-Based Care Management Program for Individuals With Anxiety Disorders
http://data.linkedct.org/resource/trials/NCT00052078	Anxiety Disorders	Child and Adolescent Anxiety Disorders (CAMS)
http://data.linkedct.org/resource/trials/NCT00248612	Anxiety Disorders	Psychosocial and Medication Treatment for Anxiety in Alcoholism
http://data.linkedct.org/resource/trials/NCT00118001	Anxiety Disorders	Psychotherapy of Generalized Anxiety Disorder
http://data.linkedct.org/resource/trials/NCT00149760	Anxiety Disorders	Effectiveness of Cognitive-Affective Behavior Therapy for the Treatment of Somatization Disorder
http://data.linkedct.org/resource/trials/NCT00280670	Anxiety Disorders	CBT for Comorbid Anxiety Disorders in Children With Autism, Asperger Syndrome, or PDD-NOS
http://data.linkedct.org/resource/trials/NCT00557648	Anxiety Disorders	Evaluating the Effectiveness of Early Cognitive Behavioral Therapy With or Without Parental Involvement in Treating Children With Anxiety Disorders
http://data.linkedct.org/resource/trials/NCT00446407	Anxiety Disorders	Evaluating the Benefits and Affordability of a Program to Improve the Care of Common Mental Disorders in Primary Care
http://data.linkedct.org/resource/trials/NCT00395213	Anxiety Disorders	Antidepressant Safety in Kids Study
http://data.linkedct.org/resource/trials/NCT00384332	Bipolar Depression	Medication, Weight Gain and GI Hormones
http://data.linkedct.org/resource/trials/NCT00457054	Bipolar Depression	Functional Connectivity in Mood Regulating Circuit In

Supplement 01 (v03) to "The Translational Medicine Ontology and Knowledge Base: Driving personalized medicine by bridging the gap between bench and bedside", Joanne S. Luciano et al., JBMS, Bio-Ontologies 2010 (2011)

		Bipolar Depression and Mania
http://data.linkedct.org/resource/trials/NCT00544544	Bipolar Depression	Riluzole in the Treatment of Bipolar Depression
http://data.linkedct.org/resource/trials/NCT00562861	Bipolar Depression	Treatment for Bipolar Depression: Acute & Prophylactic Efficacy With Citalopram
http://data.linkedct.org/resource/trials/NCT00204503	Bipolar I or II Depression and Alcohol Abuse or Dependence	Open-Label Depakote ER in Patients With Bipolar I or II Depression and Alcohol Abuse or Dependence
http://data.linkedct.org/resource/trials/NCT00596986	Chronic Depression	Getting Biased Treatment Study: How Psychotherapy and Antidepressants Change Brain Activity in Chronic Depression
http://data.linkedct.org/resource/trials/NCT00259454	Dementia, Alzheimer Type	Project COPE:Managing Dementia at Home
http://data.linkedct.org/resource/trials/NCT00009191	Depression	The Depression in Alzheimer's Disease Study (DIADS)
http://data.linkedct.org/resource/trials/NCT00019643	Depression	Factors Affecting Weight Gain in Women Receiving Adjuvant Chemotherapy for Breast Cancer
http://data.linkedct.org/resource/trials/NCT00037024	Depression	Study of Health Promotion in Patients With Early-Stage Breast or Prostate Cancer
http://data.linkedct.org/resource/trials/NCT00043550	Depression	Treatments for Depression: Drug Versus Psychotherapy
http://data.linkedct.org/resource/trials/NCT00052091	Depression	Therapy for Depressed Elders With Thought Problems
http://data.linkedct.org/resource/trials/NCT00057551	Depression	Research Evaluating the Value of Augmenting Medication With Psychotherapy (REVAMP)
http://data.linkedct.org/resource/trials/NCT00061698	Depression	Treatment for Depressed Preadolescent Girls
http://data.linkedct.org/resource/trials/NCT00068458	Depression	Survival TRaining for ENhancing Total Health (STRENGTH): Distance Medicine-Based Exercise and Diet in Preventing Body Composition Change in Women Receiving

Supplement 01 (v03) to "The Translational Medicine Ontology and Knowledge Base: Driving personalized medicine by bridging the gap between bench and bedside", Joanne S. Luciano et al., JBMS, Bio-Ontologies 2010 (2011)

		Chemotherapy for Stage I, Stage II, or Stage IIIA Breast Cancer
http://data.linkedct.org/resource/trials/NCT00112515	Depression	Modafinil in Treating Fatigue in Patients With Cancer
http://data.linkedct.org/resource/trials/NCT00122031	Depression	Deep Brain Stimulation for Treatment-Refractory Major Depression
http://data.linkedct.org/resource/trials/NCT00136318	Depression	S-Citalopram for the Prevention of PEGASYS-Induced Depression
http://data.linkedct.org/resource/trials/NCT00149838	Depression	Magnetic Brain Stimulation for the Treatment of Adult Depression
http://data.linkedct.org/resource/trials/NCT00166075	Depression	Mental Health Symptoms and Intimate Partner Violence
http://data.linkedct.org/resource/trials/NCT00178880	Depression	MRI Imaging of Chronobiologic Abnormalities in Depression
http://data.linkedct.org/resource/trials/NCT00183729	Depression	Memantine Treatment for Improving Rehabilitation Outcomes and Preventing Depression in Older Adults
http://data.linkedct.org/resource/trials/NCT00186056	Depression	Mifepristone in Refractory Depression
http://data.linkedct.org/resource/trials/NCT00192582	Depression	AIMHI NT Relapse Prevention Trial
http://data.linkedct.org/resource/trials/NCT00209118	Depression	Paroxetine for the Treatment of Interferon Related Side Effects for Hepatitis C
http://data.linkedct.org/resource/trials/NCT00214682	Depression	Beyond Ageing Project: A Study for the Prevention of Depression
http://data.linkedct.org/resource/trials/NCT00231959	Depression	Effectiveness of Pramipexole for Treatment-Resistant Depression
http://data.linkedct.org/resource/trials/NCT00234312	Depression	Medications for the Treatment of Dysthymic Disorder and Double Depression
http://data.linkedct.org/resource/trials/NCT00242619	Depression	Insulin Resistance in Patients With Mood Disorder
http://data.linkedct.org/resource/trials/NCT00280319	Depression	Study of Treatments for Depression Among

Supplement 01 (v03) to "The Translational Medicine Ontology and Knowledge Base: Driving personalized medicine by bridging the gap between bench and bedside", Joanne S. Luciano et al., JBMS, Bio-Ontologies 2010 (2011)

		Displaced Adolescents in Northern Uganda.
http://data.linkedct.org/resource/trials/NCT00298805	Depression	A Quality of Life Study in Patients With Migraines
http://data.linkedct.org/resource/trials/NCT00316303	Depression	Effectiveness of the Screen, Test, Immunize, Reduce Risk, and Refer (STIRR) Intervention for People With Both a Mental and Substance Abuse Disorder
http://data.linkedct.org/resource/trials/NCT00026637	Depression	Depression and Health Outcomes in Refractory Epilepsy
http://data.linkedct.org/resource/trials/NCT00044174	Depression	Dyadic Interactions in Depressed and Non-Depressed Mothers and Their Infants
http://data.linkedct.org/resource/trials/NCT00057577	Depression	Preventing the Recurrence of Depression With Drugs and Psychotherapy
http://data.linkedct.org/resource/trials/NCT00071162	Depression	Genetics of Fibromyalgia
http://data.linkedct.org/resource/trials/NCT00076245	Depression	Cognitive Behavioral Therapy for the Treatment of Seasonal Affective Disorder (SAD)
http://data.linkedct.org/resource/trials/NCT00086138	Depression	Depression in Alzheimer's Disease-2
http://data.linkedct.org/resource/trials/NCT00103415	Depression	Trial Investigating the Effect of Different Exercise Forms on Depression
http://data.linkedct.org/resource/trials/NCT00105573	Depression	Interventions for Depressed Low Income Mothers and Their Infants
http://data.linkedct.org/resource/trials/NCT00116857	Depression	Omega-3 Fatty Acids to Improve Depression and Reduce Cardiovascular Risk Factors
http://data.linkedct.org/resource/trials/NCT00129480	Depression	Improving Chronic Pain Treatment in Primary Care
http://data.linkedct.org/resource/trials/NCT00162916	Depression	Antidepressant Maintenance in Traumatic Brain Injury
http://data.linkedct.org/resource/trials/NCT00164203	Depression	The Feelings Club: Evaluating a School-Based Intervention for Children at Risk for Depression and Anxiety Disorders

Supplement 01 (v03) to "The Translational Medicine Ontology and Knowledge Base: Driving personalized medicine by bridging the gap between bench and bedside", Joanne S. Luciano et al., JBMS, Bio-Ontologies 2010 (2011)

http://data.linkedct.org/resource/trials/NCT00169286	Depression	Organized Self-Management Support for Chronic Depression
http://data.linkedct.org/resource/trials/NCT00178828	Depression	Dynamic Measures of Neurochemistry in Mood Disorders
http://data.linkedct.org/resource/trials/NCT00183417	Depression	Depression Prevention Program for Adolescents
http://data.linkedct.org/resource/trials/NCT00183560	Depression	Preventing Depression Relapse With Mindfulness-Based Cognitive Therapy
http://data.linkedct.org/resource/trials/NCT00183677	Depression	Brain Energy Metabolism in Individuals With Major Depressive Disorder Receiving Escitalopram
http://data.linkedct.org/resource/trials/NCT00183716	Depression	The Trauma Recovery and Empowerment Model for Treating Post-Traumatic Stress Disorder in Women
http://data.linkedct.org/resource/trials/NCT00218764	Depression	Cognitive Therapy Versus Medication Treatment for Preventing Depression Relapse
http://data.linkedct.org/resource/trials/NCT00241761	Depression	Epidemiology of Depression and Heart Failure in Aging
http://data.linkedct.org/resource/trials/NCT00245817	Depression	HINCAB-Study. Home Based Intervention in Nursing for Coronary Artery Bypass Patients
http://data.linkedct.org/resource/trials/NCT00247624	Depression	Improving Sleep and Psychological Functioning in People With Depression and Insomnia
http://data.linkedct.org/resource/trials/NCT00254007	Depression	Depression and Traumatic Brain Injury
http://data.linkedct.org/resource/trials/NCT00258752	Depression	Comparison of Three Therapy-Based Interventions for Preventing Depression in Adolescents
http://data.linkedct.org/resource/trials/NCT00285584	Depression	Bupropion For Reducing High-Risk Behaviors in Depressed Men Who Have Sex With Men (MSM)
http://data.linkedct.org/resource/trials/NCT00304915	Depression	HIV Translating Initiatives for Depression Into Effective Solutions
http://data.linkedct.org/resource	Depression	Study Comparing

Supplement 01 (v03) to "The Translational Medicine Ontology and Knowledge Base: Driving personalized medicine by bridging the gap between bench and bedside", Joanne S. Luciano et al., JBMS, Bio-Ontologies 2010 (2011)

/trials/NCT00305565		Outcomes for Patients With Treatment Resistant Depression Who Receive VNS Therapy at Different Doses
http://data.linkedct.org/resource/trials/NCT00338962	Depression	Naltrexone & SSRI in Alcoholics With Depression/PTSD
http://data.linkedct.org/resource/trials/NCT00027378	Depression	Pharmacological Intervention Project (Fluoxetine)
http://data.linkedct.org/resource/trials/NCT00029172	Depression	Treatment for Post-Stroke Depression
http://data.linkedct.org/resource/trials/NCT00071643	Depression	Preventing Post-Stroke Depression
http://data.linkedct.org/resource/trials/NCT00073359	Depression	Effects of Therapist Behavior on the Treatment of Depressed Adolescents
http://data.linkedct.org/resource/trials/NCT00073671	Depression	Prevention of Depression in At-Risk Adolescents
http://data.linkedct.org/resource/trials/NCT00074789	Depression	Reducing Symptoms of Depression in Low-Income Mothers
http://data.linkedct.org/resource/trials/NCT00076258	Depression	Adding Exercise to Antidepressant Medication Treatment for Depression
http://data.linkedct.org/resource/trials/NCT00076622	Depression	Medication Treatment for Depression in Nursing Home Residents
http://data.linkedct.org/resource/trials/NCT00091962	Depression	Effectiveness of Depression Treatment Following Coronary Artery Bypass Surgery
http://data.linkedct.org/resource/trials/NCT00118404	Depression	Cognitive Therapy for Recurrent Depression
http://data.linkedct.org/resource/trials/NCT00118430	Depression	Stepped Care for Depression and Musculoskeletal Pain
http://data.linkedct.org/resource/trials/NCT00140257	Depression	DECARD: Study of Escitalopram in the Prevention of Depression in Patients With Acute Coronary Syndrome
http://data.linkedct.org/resource/trials/NCT00152763	Depression	Psychological Support for Patients With an Implantable Cardioverter Defibrillator
http://data.linkedct.org/resource/trials/NCT00159965	Depression	Treatments for Psychogenic Nonepileptic

Supplement 01 (v03) to "The Translational Medicine Ontology and Knowledge Base: Driving personalized medicine by bridging the gap between bench and bedside", Joanne S. Luciano et al., JBMS, Bio-Ontologies 2010 (2011)

		Seizures (NES)
http://data.linkedct.org/resource/trials/NCT00177294	Depression	Augmenting Antidepressant Treatment With Interpersonal Psychotherapy for Treating Late-Life Depression
http://data.linkedct.org/resource/trials/NCT00177671	Depression	Antidepressant Medication Plus Donepezil for Treating Late-Life Depression
http://data.linkedct.org/resource/trials/NCT00178906	Depression	Acoustical Properties of Speech as Indicators of Suicidal Risk
http://data.linkedct.org/resource/trials/NCT00181961	Depression	Safety and Effectiveness Study of Maca Root to Treat Antidepressant-Induced Sexual Dysfunction
http://data.linkedct.org/resource/trials/NCT00182078	Depression	A Study of Sertraline to Prevent PTSD
http://data.linkedct.org/resource/trials/NCT00183326	Depression	Treating Post Traumatic Stress Disorder in Children Exposed to Domestic Violence
http://data.linkedct.org/resource/trials/NCT00206362	Depression	Ability to Cope With Type 1 Diabetes
http://data.linkedct.org/resource/trials/NCT00223652	Depression	Telephone Administered Psychotherapy for the Treatment of Depression for Veterans in Rural Areas
http://data.linkedct.org/resource/trials/NCT00226642	Depression	Treatment of Mild and Subthreshold Depressive Disorders
http://data.linkedct.org/resource/trials/NCT00241774	Depression	Coronary Heart Disease Incidence: Depression & Inflammation Risk
http://data.linkedct.org/resource/trials/NCT00279370	Depression	Antidepressant Use During Pregnancy
http://data.linkedct.org/resource/trials/NCT00285818	Depression	Mifepristone Used to Treat Patients With Non-Psychotic Major Depressive Disorder Referred for Bilateral Electroconvulsive Therapy (ECT)
http://data.linkedct.org/resource/trials/NCT00316290	Depression	Integrated Parent Training for Treating Depression in Mothers of Children With Attention Deficit Hyperactivity Disorder

Supplement 01 (v03) to "The Translational Medicine Ontology and Knowledge Base: Driving personalized medicine by bridging the gap between bench and bedside", Joanne S. Luciano et al., JBMS, Bio-Ontologies 2010 (2011)

http://data.linkedct.org/resource/trials/NCT00344474	Depression	Personality-Targeted Interventions for Adolescent Alcohol Misuse
http://data.linkedct.org/resource/trials/NCT00360451	Depression	Effectiveness of School-Based Cognitive Behavioral Therapy in Preventing Depression in Young Adolescents
http://data.linkedct.org/resource/trials/NCT00381823	Depression	Healthy Outcomes of Pregnancy Education
http://data.linkedct.org/resource/trials/NCT00430404	Depression	Screening and Treatment of Depression in the Community
http://data.linkedct.org/resource/trials/NCT00468676	Depression	Nurse-Led Case Management for Diabetes and Cardiovascular Disease Patients With Depression
http://data.linkedct.org/resource/trials/NCT00582595	Depression	LABS-3 Psychosocial
http://data.linkedct.org/resource/trials/NCT00601393	Depression	Effectiveness of Internet-Based Cognitive Behavioral Therapy for Treating Mothers With Depression
http://data.linkedct.org/resource/trials/NCT00619619	Depression	Study Evaluating Desvenlafaxine Succinate Sustained-Release (DVS SR) in the Treatment of Child and Adolescent Outpatients With Major Depressive Disorder
http://data.linkedct.org/resource/trials/NCT00624013	Depression	Diabetes and Depression in Hispanics and African Americans: Treatment of Depression With Sertraline and Its Effect on A1c and Quality of Life
http://data.linkedct.org/resource/trials/NCT00664885	Depression	Effectiveness of Cultural Sensitive Collaborative Treatment of Depressed Chinese
http://data.linkedct.org/resource/trials/NCT00672880	Depression	Self System Therapy and Chronic Low Back Pain Trial
http://data.linkedct.org/resource/trials/NCT00676962	Depression	Facilitating Implementation of Cognitive Behavioral Therapy in Primary Care and Community Clinics

http://data.linkedct.org/resource/trials/NCT00373022	Depression	Moderate Physical Activity in Helping Patients Recover Physically and Emotionally From Stage II Or Stage III Colorectal Cancer
http://data.linkedct.org/resource/trials/NCT00416221	Depression	PACEPro - Mood Management Pilot
http://data.linkedct.org/resource/trials/NCT00436150	Depression	Interpersonal Therapy-Based Treatment to Prevent Postpartum Depression in Adolescent Mothers
http://data.linkedct.org/resource/trials/NCT00446407	Depression	Evaluating the Benefits and Affordability of a Program to Improve the Care of Common Mental Disorders in Primary Care
http://data.linkedct.org/resource/trials/NCT00449007	Depression	Fluoxetine and Bupropion to Treat Patients With Depression and Alcoholism
http://data.linkedct.org/resource/trials/NCT00459329	Depression	PEARL Intervention to Reduce Depression Among Adults With Epilepsy
http://data.linkedct.org/resource/trials/NCT00495768	Depression	Reducing Depressive Symptoms During HCV Therapy: A Randomized Study
http://data.linkedct.org/resource/trials/NCT00499759	Depression	Individualized Exercise Programs for Patients Receiving Chemotherapy for Newly Diagnosed Acute or Relapsed Myeloid Leukemia or Lymphoblastic Leukemia
http://data.linkedct.org/resource/trials/NCT00611975	Depression	Effect of Antidepressants on Sex Hormone Levels and Sexual Functioning
http://data.linkedct.org/resource/trials/NCT00456430	Depression	Serotonin Transporter Genetic Variation and Amygdalar Activation Correlates of Antidepressant Response
http://data.linkedct.org/resource/trials/NCT00499785	Depression	Physical and Mental Assessment of Older Patients With Newly Diagnosed Acute Myeloid Leukemia

Supplement 01 (v03) to "The Translational Medicine Ontology and Knowledge Base: Driving personalized medicine by bridging the gap between bench and bedside", Joanne S. Luciano et al., JBMS, Bio-Ontologies 2010 (2011)

http://data.linkedct.org/resource/trials/NCT00573547	Depression	Open Label Trial of Fluoxetine for the Treatment of Depression in Patients With End Stage Renal Disease
http://data.linkedct.org/resource/trials/NCT00574145	Depression	Healing Touch in Treating Fatigue in Women Undergoing Radiation Therapy for Breast Cancer
http://data.linkedct.org/resource/trials/NCT00578318	Depression	African American Knowledge Optimized for Mindfully Healthy Adolescents
http://data.linkedct.org/resource/trials/NCT00594711	Depression	Monoamine Transporter Function and Autonomic Regulation During Psychodynamic Psychotherapy
http://data.linkedct.org/resource/trials/NCT00634608	Depression	Integrating Targeted MedlinePlus Health Prescriptions Into Clinic Practice Workflow
http://data.linkedct.org/resource/trials/NCT00640562	Depression	Quetiapine Extended Release Depression Symptoms
http://data.linkedct.org/resource/trials/NCT00641940	Depression	Girls in Transition Study: Helping Girls Enter the Teenage Years
http://data.linkedct.org/resource/trials/NCT00669110	Depression	Study Evaluating DVS SR in the Treatment of Child and Adolescent Outpatients With Major Depressive Disorder
http://data.linkedct.org/resource/trials/NCT00694681	Depression	Working Toward Wellness
http://data.linkedct.org/resource/trials/NCT00709150	Depression	Effectiveness of Collaborative Depression Care Management in Treating Depressed Low-Income Hispanics With Diabetes
http://data.linkedct.org/resource/trials/NCT00770068	Depression	Examining the Link Between Depression and Seasonal Allergies
http://data.linkedct.org/resource/trials/NCT00776685	Depression	Adventure: The Efficacy of Personality-Targeted Interventions for Substance Misuse and Other Risky Behaviours as Delivered by

		Educational Professionals
http://data.linkedct.org/resource/trials/NCT00785603	Depression	Paroxetine Effect on Tramadol Metabolism and Pharmacodynamics: a Dose Response Study
http://data.linkedct.org/resource/trials/NCT00906581	Depression	Self-Guided Depression Treatment on Long-Duration Spaceflights: A Continuation Study
http://data.linkedct.org/resource/trials/NCT00788814	Depression	Study of Pathways Curriculum for Use With WPD
http://data.linkedct.org/resource/trials/NCT00845455	Depression	Personality Type as a Predictor to Develop Depression and Reduction in Quality of Life Among Stroke Survivors.
http://data.linkedct.org/resource/trials/NCT00863291	Depression	A Double-Blind Study of Buprenorphine Treatment of Acute Suicidality
http://data.linkedct.org/resource/trials/NCT00186186	Depression, Bipolar	Depakote ER in Bipolar Depression
http://data.linkedct.org/resource/trials/NCT00490971	Depression, Bipolar	A Study to Evaluate the Effectiveness and Safety of Extended-Release (ER) Paliperidone Compared With Placebo in Delaying the Recurrence of Symptoms in Bipolar I Disorder
http://data.linkedct.org/resource/trials/NCT00510146	Depression, Bipolar	Olanzapine Treatment of Patients With Bipolar I Disorder
http://data.linkedct.org/resource/trials/NCT00051246	Depression, Postpartum	Group Therapy for Postpartum Depression
http://data.linkedct.org/resource/trials/NCT00219349	Generalized Anxiety Disorder	Cognitive-Behavioral Therapy and Lexapro for GAD
http://data.linkedct.org/resource/trials/NCT00347269	Generalized Anxiety Disorder	Primary Care Intervention Strategy for Anxiety Disorders
http://data.linkedct.org/resource/trials/NCT00052078	Generalized Anxiety Disorder	Child and Adolescent Anxiety Disorders (CAMS)
http://data.linkedct.org/resource/trials/NCT00120458	Generalized Anxiety Disorder	Black Cohosh as Alternative Therapy for Treating Menopause-Related Anxiety
http://data.linkedct.org/resource/trials/NCT00308724	Generalized Anxiety Disorder	Treating Late-Life Generalized Anxiety

Supplement 01 (v03) to "The Translational Medicine Ontology and Knowledge Base: Driving personalized medicine by bridging the gap between bench and bedside", Joanne S. Luciano et al., JBMS, Bio-Ontologies 2010 (2011)

		Disorder (GAD) in Primary Care
http://data.linkedct.org/resource/trials/NCT00620776	Generalized Anxiety Disorder	Combined Treatment for Generalized Anxiety Disorder (GAD)
http://data.linkedct.org/resource/trials/NCT00569829	Generalized Anxiety Disorder	Modular Cognitive Behavioral Therapy for the Treatment of Child Anxiety Disorders in Elementary School Settings
http://data.linkedct.org/resource/trials/NCT00515242	Generalized Anxiety Disorder	Therapeutic Massage for Generalized Anxiety Disorder
http://data.linkedct.org/resource/trials/NCT00788034	Generalized Anxiety Disorder	Relapse-prevention Study With Lu AA21004 in Patients With Generalized Anxiety Disorder
http://data.linkedct.org/resource/trials/NCT00803361	Generalized Anxiety Disorder	Duloxetine for the Treatment of Generalized Anxiety Disorder
http://data.linkedct.org/resource/trials/NCT00902564	Generalized Anxiety Disorder	Efficacy and Safety of Escitalopram in Patients With Generalized Anxiety Disorder
http://data.linkedct.org/resource/trials/NCT00174577	Major Depression	Risperidone Augmentation Therapy in Patients Who Have Failed or Only Partially Responded to a Trial of Antidepressant
http://data.linkedct.org/resource/trials/NCT00291239	Major Depression	Effect of Partial Sleep Deprivation on Cognition and Cytokines in Individuals With Major Depression
http://data.linkedct.org/resource/trials/NCT00616759	Major Depression	The Effect on Cognition of Terminating ECT Induced Seizures With Propofol
http://data.linkedct.org/resource/trials/NCT00461279	Major Depression	Attachment Security as Mediator and Moderator of Outcome in Major Depression
http://data.linkedct.org/resource/trials/NCT00532480	Major Depression	Study of Brain Response to Emotional Pictures Using a fMRI While on Duloxetine
http://data.linkedct.org/resource/trials/NCT00627029	Major Depression	Evaluation of Programs of Coordinated Care and Disease Management
http://data.linkedct.org/resource/trials/NCT00472576	Major Depression	Antidepressant Effects of NR2B in Major Depression

Supplement 01 (v03) to "The Translational Medicine Ontology and Knowledge Base: Driving personalized medicine by bridging the gap between bench and bedside", Joanne S. Luciano et al., JBMS, Bio-Ontologies 2010 (2011)

http://data.linkedct.org/resource/trials/NCT00541957	Major Depression	Randomized Trial of Psychotherapy for Persistently Depressed Primary Care Patients
http://data.linkedct.org/resource/trials/NCT00565110	Major Depression	Treating Depression Among Low-Income Patients With Cancer
http://data.linkedct.org/resource/trials/NCT00918684	Major Depression	Aging Brain Changes, Executive Dysfunction and Depression
http://data.linkedct.org/resource/trials/NCT00243451	Mild Alzheimer's Disease	Early Detection of Mild Cognitive Impairment in Individual Patients
http://data.linkedct.org/resource/trials/NCT00595582	Mild Alzheimer's Disease	Early Intervention in Mild Cognitive Impairment (MCI) With Curcumin + Bioperine
http://data.linkedct.org/resource/trials/NCT00571064	Mild to Severe Alzheimer's Disease	The Effectiveness And Safety Of Donepezil Hydrochloride (E2020) In Subjects With Mild To Severe Alzheimer's Disease Residing In An Assisted Living Facility
http://data.linkedct.org/resource/trials/NCT00692432	Myocardial Depression	Macrophage Inhibitory Factor (MIF) and High-Mobility Group-1 Protein (HMG-1) in Children Undergoing Cardiopulmonary Bypass
http://data.linkedct.org/resource/trials/NCT00025766	Myocardial Infarction	Angioplasty and Heart Stents to Treat Individuals With an Occluded Artery Following a Heart Attack
http://data.linkedct.org/resource/trials/NCT00066898	Myocardial Infarction	ARISE – Aggressive Reduction of Inflammation Stops Events
http://data.linkedct.org/resource/trials/NCT00075088	Myocardial Infarction	Tele-Electrocardiography in Emergency Cardiac Care
http://data.linkedct.org/resource/trials/NCT00114452	Myocardial Infarction	Safety Study of Adult Mesenchymal Stem Cells (MSC) to Treat Acute Myocardial Infarction
http://data.linkedct.org/resource/trials/NCT00119847	Myocardial Infarction	Balloon Catheters and Stents to Prevent Heart Rhythm Irregularities in Individuals Post-Heart Attack

http://data.linkedct.org/resource/trials/NCT00126750	Myocardial Infarction	Web-Enhanced Guideline Implementation for Post MI CBOC Patients
http://data.linkedct.org/resource/trials/NCT00164190	Myocardial Infarction	Routine Angioplasty and Stenting After Fibrinolysis for Acute Myocardial Infarction
http://data.linkedct.org/resource/trials/NCT00195169	Myocardial Infarction	Quality of Life After Myocardial Infarction
http://data.linkedct.org/resource/trials/NCT00253097	Myocardial Infarction	The Post Stroke Preventive Trial (PREVENT). A RCT Nested in a Cohort Study
http://data.linkedct.org/resource/trials/NCT00269425	Myocardial Infarction	The Heart Institute of Spokane Diet Study
http://data.linkedct.org/resource/trials/NCT00303979	Myocardial Infarction	IMPROVE HF: Registry to Improve the Use of Evidence-Based Heart Failure Therapies in the Outpatient Setting
http://data.linkedct.org/resource/trials/NCT00004562	Myocardial Infarction	Occluded Artery Trial (OAT)
http://data.linkedct.org/resource/trials/NCT00013741	Myocardial Infarction	The MI-HEART Project
http://data.linkedct.org/resource/trials/NCT00066053	Myocardial Infarction	Periodontitis and Cardiovascular Events or "PAVE"
http://data.linkedct.org/resource/trials/NCT00099411	Myocardial Infarction	Heart Muscle Viability and Remodeling in Individuals Post-Heart Attack
http://data.linkedct.org/resource/trials/NCT00116857	Myocardial Infarction	Omega-3 Fatty Acids to Improve Depression and Reduce Cardiovascular Risk Factors
http://data.linkedct.org/resource/trials/NCT00139464	Myocardial Infarction	Alpha Omega Trial
http://data.linkedct.org/resource/trials/NCT00149591	Myocardial Infarction	The Viability-Guided Angioplasty After Acute Myocardial Infarction-Trial (The VIAMI-Trial)
http://data.linkedct.org/resource/trials/NCT00175058	Myocardial Infarction	Acute Myocardial Infarction With HyperOxemic Therapy II (AMIHOT II)
http://data.linkedct.org/resource/trials/NCT00279175	Myocardial Infarction	REPAIR-AMI: Intracoronary Progenitor Cells in Acute Myocardial Infarction (AMI)
http://data.linkedct.org/resource/trials/NCT00005487	Myocardial Infarction	Multi-Ethnic Study of Atherosclerosis (MESA)
http://data.linkedct.org/resource	Myocardial Infarction	Look AHEAD: Action for

Supplement 01 (v03) to "The Translational Medicine Ontology and Knowledge Base: Driving personalized medicine by bridging the gap between bench and bedside", Joanne S. Luciano et al., JBMS, Bio-Ontologies 2010 (2011)

/trials/NCT00017953		Health in Diabetes
http://data.linkedct.org/resource/trials/NCT00054678	Myocardial Infarction	MYOHEART™ (Myogenesis Heart Efficiency and Regeneration Trial)
http://data.linkedct.org/resource/trials/NCT00071032	Myocardial Infarction	Safety and Effectiveness of Two Blood Transfusion Strategies in Surgical Patients With Cardiovascular Disease
http://data.linkedct.org/resource/trials/NCT00093197	Myocardial Infarction	Safety of KAI-9803 for Injection With Angioplasty Following Heart Attack
http://data.linkedct.org/resource/trials/NCT00106886	Myocardial Infarction	HOPE-2 Study (Heart Outcomes Prevention Evaluation-2 Study)
http://data.linkedct.org/resource/trials/NCT00128024	Myocardial Infarction	Effects of Early Statin Treatment After Acute Myocardial Infarction (AMI) in Japanese Patients
http://data.linkedct.org/resource/trials/NCT00136929	Myocardial Infarction	Senior PAMI: Primary Angioplasty Versus Thrombolytic Therapy for Acute Myocardial Infarction in the Elderly
http://data.linkedct.org/resource/trials/NCT00229515	Myocardial Infarction	Multicentre 2x2 Factorial Randomised Study Comparing Tirofiban Versus Abciximab and SES Versus BMS in AMI
http://data.linkedct.org/resource/trials/NCT00235339	Myocardial Infarction	Effect of Standard Care Rehabilitation Versus Interval Treadmill Training After Myocardial Infarction
http://data.linkedct.org/resource/trials/NCT00288210	Myocardial Infarction	Sirolimus Eluting Stenting in Acute Myocardial Infarction
http://data.linkedct.org/resource/trials/NCT00336830	Myocardial Infarction	Improving Cardiac Rehabilitation Participation in Women and Men
http://data.linkedct.org/resource/trials/NCT00375817	Myocardial Infarction	Safety and Effects of Implanted (Autologous) Skeletal Myoblasts (MyoCell) Using an Injection Catheter = SEISMIC Trial
http://data.linkedct.org/resource/trials/NCT00395811	Myocardial Infarction	Stem Cell Therapy to Improve Myocardial Function in Patients

Supplement 01 (v03) to "The Translational Medicine Ontology and Knowledge Base: Driving personalized medicine by bridging the gap between bench and bedside", Joanne S. Luciano et al., JBMS, Bio-Ontologies 2010 (2011)

		Undergoing Coronary Artery Bypass Grafting (CABG)
http://data.linkedct.org/resource/trials/NCT00426231	Myocardial Infarction	Culturally-Tailored Approach to Improve Medication Use in Patients With Heart Attacks
http://data.linkedct.org/resource/trials/NCT00617123	Myocardial Infarction	Trial to Assess the Ocular Safety of SCH 530348 in Patients With Atherosclerosis (Study P05183AM1)
http://data.linkedct.org/resource/trials/NCT00638638	Myocardial Infarction	Myocardial Infarction With ST-Elevation
http://data.linkedct.org/resource/trials/NCT00389545	Myocardial Infarction	Intracoronary Stem Cells in Large Myocardial Infarction
http://data.linkedct.org/resource/trials/NCT00403728	Myocardial Infarction	Ciclosporin A and Acute Myocardial Infarction
http://data.linkedct.org/resource/trials/NCT00430820	Myocardial Infarction	Discovery of New Circulating Biomarkers of Coronary Atherosclerosis
http://data.linkedct.org/resource/trials/NCT00489827	Myocardial Infarction	Glutamate for Metabolic Intervention in Coronary Surgery
http://data.linkedct.org/resource/trials/NCT00590291	Myocardial Infarction	Molecular Determinants of Coronary Artery Disease
http://data.linkedct.org/resource/trials/NCT00692991	Myocardial Infarction	Using Magnetic Resonance Imaging to Evaluate Heart Vessel Function After Angioplasty or Stent Placement Procedures
http://data.linkedct.org/resource/trials/NCT00433966	Myocardial Infarction	Harmonizing Outcomes With Revascularization and Stents in Acute Myocardial Infarction
http://data.linkedct.org/resource/trials/NCT00442806	Myocardial Infarction	Randomized Clinical Trial of Adipose-Derived Stem Cells in the Treatment of Pts With ST-Elevation Myocardial Infarction
http://data.linkedct.org/resource/trials/NCT00526474	Myocardial Infarction	Trial to Assess the Effects of SCH 530348 in Preventing Heart Attack and Stroke in Patients With Atherosclerosis (TRA 2°P - TIMI 50) (Study P04737AM2)
http://data.linkedct.org/resource	Myocardial Infarction	Study of Myocardial

Supplement 01 (v03) to "The Translational Medicine Ontology and Knowledge Base: Driving personalized medicine by bridging the gap between bench and bedside", Joanne S. Luciano et al., JBMS, Bio-Ontologies 2010 (2011)

/trials/NCT00575289		Perfusion and Coronary Anatomy Imaging Roles in Coronary Artery Disease
http://data.linkedct.org/resource/trials/NCT00626145	Myocardial Infarction	Long Term Follow-up of Autologous Bone Marrow Mononuclear Cells Therapy in STEMI
http://data.linkedct.org/resource/trials/NCT00671346	Myocardial Infarction	NORVIT and WENBIT - Long-term Follow-up
http://data.linkedct.org/resource/trials/NCT00691470	Myocardial Infarction	Comparison of ATI-5923, a Novel Vitamin K Antagonist, With Warfarin in Patients Requiring Chronic Anticoagulation
http://data.linkedct.org/resource/trials/NCT00872456	Myocardial Infarction	Study to Asses the Incidence of Cardiovascular Disease (CVD) Complications in Diabetic Patients Pre-Stratified by Haptoglobin Phenotype and HbA1c From the I CARE Registry
http://data.linkedct.org/resource/trials/NCT00926133	Myocardial Infarction	Type 2 Diabetes and Acute Myocardial Infarction
http://data.linkedct.org/resource/trials/NCT00936169	Myocardial Infarction	Angiography Versus (vs) IVUS Optimisation
http://data.linkedct.org/resource/trials/NCT00863967	Myocardial Infarction	Early Detection of Arteriosclerosis
http://data.linkedct.org/resource/trials/NCT00846222	Myocardial Infarction Complicated With Cardiogenic Shock	Adjunctive Mild Hypothermia Therapy to Primary Percutaneous Coronary Intervention in Patients With Acute Myocardial Infarction Complicated With Shock: A Feasibility Study
http://data.linkedct.org/resource/trials/NCT00842946	Nongeneralized Social Anxiety Disorder	Exposure With Acceptance-Based Versus Habituation-Based Rationale for Public Speaking Anxiety
http://data.linkedct.org/resource/trials/NCT00605631	Post Myocardial Infarction	The Prevention of Myocardial Enlargement and Dilatation Post Myocardial Infarction Study
http://data.linkedct.org/resource/trials/NCT00360204	Postpartum Depression	Improving Health Outcomes for New Mothers and Babies
http://data.linkedct.org/resource	Postpartum Depression	Mom: Managing Our Mood,

Supplement 01 (v03) to "The Translational Medicine Ontology and Knowledge Base: Driving personalized medicine by bridging the gap between bench and bedside", Joanne S. Luciano et al., JBMS, Bio-Ontologies 2010 (2011)

/trials/NCT00795652		Part of The Family Help Program
http://data.linkedct.org/resource/trials/NCT00556140	Psychotic Depression	The Combination of Aripiprazole and Antidepressants in Psychotic Major Depression
http://data.linkedct.org/resource/trials/NCT00842946	Public Speaking Anxiety	Exposure With Acceptance-Based Versus Habituation-Based Rationale for Public Speaking Anxiety
http://data.linkedct.org/resource/trials/NCT00875134	Respiratory Depression	Testing of the Apnea Prevention Device
http://data.linkedct.org/resource/trials/NCT00502528	ST-Elevation Myocardial Infarction	Endothelin Receptor Blockade in Acute ST-elevation Myocardial Infarction
http://data.linkedct.org/resource/trials/NCT00763828	ST-Elevation Myocardial Infarction	Can Hypothermia be Incorporated Into Primary Angioplasty for Heart Attack?
http://data.linkedct.org/resource/trials/NCT00507156	ST-Segment Myocardial Infarction	Postconditioning in the Treatment of Acute ST-Segment Elevation Myocardial Infarction
http://data.linkedct.org/resource/trials/NCT00569829	Separation Anxiety Disorder	Modular Cognitive Behavioral Therapy for the Treatment of Child Anxiety Disorders in Elementary School Settings
http://data.linkedct.org/resource/trials/NCT00347269	Social Anxiety Disorder	Primary Care Intervention Strategy for Anxiety Disorders
http://data.linkedct.org/resource/trials/NCT00902226	Social Anxiety Disorder	Safety of Escitalopram in Patients With Social Anxiety Disorder
http://data.linkedct.org/resource/trials/NCT00565617	Treatment Resistant Depression	Epidural Cortical Stimulation for Depression
http://data.linkedct.org/resource/trials/NCT00322777	Unipolar Depression	Spirituality and Depression Study

-----

Q8. Do I have suitable patients for an AD trial where they are looking for females who are aged over 55 years, have the APOE variant, and low ADAS COG scores?

SPARQL

Supplement 01 (v03) to "The Translational Medicine Ontology and Knowledge Base: Driving personalized medicine by bridging the gap between bench and bedside", Joanne S. Luciano et al., JBMS, Bio-Ontologies 2010 (2011)

PREFIX indivo: <http://indivo.org/vocab/xml/documents#>
 PREFIX trans: <tag:eric@w3.org:2009/tmo/translator#>
 PREFIX foaf: <http://xmlns.com/foaf/0.1/>

```
SELECT DISTINCT(?patient_name) ?dob ?gender
WHERE {
  GRAPH <pchr> {
    ?patient foaf:name ?patient_name .
    ?patient trans:demographics ?demographics .
    # female
    ?demographics indivo:gender ?gender .
    FILTER ( ?gender = "Female" ) .
    # age > 55 ; would have been born in 1954
    ?demographics indivo:dateOfBirth ?dob .
    FILTER ( ?dob < "1954-01-01" )
    # apoe variant
    [] trans:patient ?patient ;
      trans:test [ trans:result [ trans:variant_Synonyms ?synonyms ] ] .
    FILTER regex(?synonyms, "APOE4") .
    # low ADAS COG score: Alzheimer's Disease Assessment Scale-cognitive subscale
    # trans:testName "Alzheimer's Disease Assessment Scale-cognitive subscale" ;
    #[] trans:patient ?patient ;
    #   trans:test [ trans:CPT "97532" ; trans:result [ ?p ?stuff ] ] .
  }
}
```

Results

patient_name	dob	gender
Marianne Huber	1940-01-01	Female
Monica Mary Mall	1937-05-15	Female

Q9. What genes are associated with or implicated in AD?

SPARQL

PREFIX diseasome: <http://www4.wiwiwiss.fu-berlin.de/diseasome/resource/diseasome/>
 PREFIX pharmgkb: <http://bio2rdf.org/pharmgkb:>

```
SELECT DISTINCT(?gene_name)
WHERE {
  {
    GRAPH <diseasome> {
      ?disease rdfs:label ?disease_name .
      FILTER regex(?disease_name, "alzheimer", "i") .
      ?disease diseasome:associatedGene ?gene .
      ?gene rdfs:label ?gene_name .
    }
  }
}
```

Supplement 01 (v03) to "The Translational Medicine Ontology and Knowledge Base: Driving personalized medicine by bridging the gap between bench and bedside", Joanne S. Luciano et al., JBMS, Bio-Ontologies 2010 (2011)

```

} UNION {
  GRAPH <pharmgkb> {
    ?association rdf:type pharmgkb:Association .
    ?association pharmgkb:disease ?disease .
    ?disease rdfs:label ?disease_name .
    FILTER regex(?disease_name, "alzheimer", "i") .
    ?association pharmgkb:gene ?gene .
    ?gene dc:identifier ?gene_name .
  }
}
ORDER BY ASC(?gene_name)

```

Results

gene_name
A2M
AAA
ABCB4
ACE
ACE1
AD1
AD2
AD3
AD4
ADAM12
APBB2
APOE
APP
ATP8A1
BCHE
BLMH
BMH
C1QB
C1orf49
CALHM1
CBS
CD86
CHAT
CHRNA7
CLSTN2
CRP
CSF1
CST3
CTSD
CVAP
CYP2C18
CYP2C19

Supplement 01 (v03) to "The Translational Medicine Ontology and Knowledge Base: Driving personalized medicine by bridging the gap between bench and bedside", Joanne S. Luciano et al., JBMS, Bio-Ontologies 2010 (2011)

CYP2C8
CYP2C9
DCP1
DISC1
DNMBP
FE65L1
GBP2
GSTM1
GSTT1
HTR1A
HTR7
IDE
KCNMA1
KCTD12
KIF20B
LILRA3
MAPT
MEOX2
MME
MPO
NAT2
NCSTN
NMNAT3
NOS2
NOS3
OPCML
PACIP1
PAXIP1
PAXIP1L
PCDH11X
PLAU
PPP1R11
PSEN1
PSEN2
PTIP
RALGPS2
RGSL1
SIRT1
SORL1
SST
STM2
TF
UBQLN1
URK
VEGFA
WWC1
ZNF224

Supplement 01 (v03) to "The Translational Medicine Ontology and Knowledge Base: Driving personalized medicine by bridging the gap between bench and bedside", Joanne S. Luciano et al., JBMS, Bio-Ontologies 2010 (2011)

Q10. What biomarkers are associated with or implicated in AD?

SPARQL

PREFIX pharmgkb: <http://bio2rdf.org/pharmgkb:>

```
SELECT DISTINCT(?variant) ?gene_name ?description
WHERE {
  GRAPH <pharmgkb> {
    ?association a pharmgkb:DrugGeneVariantInteraction .
    ?association pharmgkb:description ?description .
    ?association pharmgkb:disease ?disease .
    ?association pharmgkb:variant ?variant .
    ?disease rdfs:label ?disease_name .
    FILTER regex(?disease_name, "alzheimer", "i") .
    ?association pharmgkb:gene ?gene .
    ?gene dc:identifier ?gene_name .
  }
}
```

ORDER BY ASC(?gene_name)

Results

variant	gene_name	description
http://bio2rdf.org/dbsnp:rs4343	ACE	In an inbred Israeli Arab community, rs4343 was shown to be significantly associated with risk of developing Alzheimer Disease, especially when analyzed in a combined haplotype with rs4351.
http://bio2rdf.org/dbsnp:rs4351	ACE	In an inbred Israeli Arab population, rs4351 was shown to be significantly associated with risk of developing Alzheimer Disease, especially when analyzed in a combined haplotype with rs4343.
http://bio2rdf.org/dbsnp:rs11244841	ADAM12	This variant is nominally associated with late-onset Alzheimer disease (LOAD) in 2 GWAS studies.
http://bio2rdf.org/dbsnp:rs2066853	AHR	Conflicting studies have researched this variant in relationship to Alzheimer's, bladder cancer and lung cancer.
http://bio2rdf.org/dbsnp:rs4420638	APOC1	GWAS results: A high-density whole-genome association study reveals that APOE is the major susceptibility gene for sporadic late-onset Alzheimer's disease. (Initial Sample Size: 664 cases, 422 controls; Replication Sample Size: NR); (Region: 19q13.32; Reported Gene(s): APOE); (p-value=

Supplement 01 (v03) to "The Translational Medicine Ontology and Knowledge Base: Driving personalized medicine by bridging the gap between bench and bedside", Joanne S. Luciano et al., JBMS, Bio-Ontologies 2010 (2011)

		9.999999999999999E-40).This variant is associated with Late onset Alzheimer's disease.
http://bio2rdf.org/dbsnp:rs4420638	APOC1	GWAS results: Candidate single-nucleotide polymorphisms from a genomewide association study of Alzheimer disease. (Initial Sample Size: 753 cases, 736 controls; Replication Sample Size: 418 cases, 249 controls); (Region: 19q13.32; Reported Gene(s): APOE, APOC; Risk Allele: rs4420638-?); (p-value=1.999999999999999E-44).This variant is associated with Alzheimer's disease.
http://bio2rdf.org/dbsnp:rs4420638	APOC1	GWAS results: Sorl1 as an Alzheimer's disease predisposition gene?. (Initial Sample Size: 664 cases, 422 controls; Replication Sample Size: NR); (Region: 19q13.32; Reported Gene(s): APOE; Risk Allele: rs4420638-?); (p-value=9.999999999999999E-40).This variant is associated with Alzheimer's disease.
http://bio2rdf.org/dbsnp:rs4420638	APOC1	In a GWAS of Alzheimer Disease families of self-reported European ancestry, this SNP was found to be associated with Alzheimer Disease.
http://bio2rdf.org/dbsnp:rs12639920	ATP8A1	This variant is significantly associated with late-onset Alzheimer disease (LOAD) in a GWAS study with 492 LOAD cases and 498 cognitive controls using Illumina's HumanHap550 beadchip.
http://bio2rdf.org/dbsnp:rs179943	ATXN1	In a GWAS of Alzheimer Disease families of self-reported European ancestry, this SNP was found to be associated with Alzheimer Disease. This association did not reach significance in replication attempts with three other family samples.
http://bio2rdf.org/dbsnp:rs2986017	CALHM1	This variant increases amyloid-beta levels by interfering with CALHM1-mediated Ca(2+) permeability. The SNP is significantly associated with Alzheimer's disease in independent case-control studies of 3404 participants.
http://bio2rdf.org/dbsnp:rs2986017	CALHM2	This variant increases amyloid-beta levels by interfering with CALHM1-mediated Ca(2+) permeability. The SNP is significantly associated with Alzheimer's disease in independent case-control studies of 3404 participants.
http://bio2rdf.org/dbsnp:rs4420638	CD33	GWAS results: Genome-wide Association

p:rs3826656		Analysis Reveals Putative Alzheimer's Disease Susceptibility Loci in Addition to APOE. (Initial Sample Size: 941 affected individuals, 404 unaffected individuals; Replication Sample Size: 1,767 affected individuals, 838 unaffected individuals); (Region: 19q13.33; Reported Gene(s): CD33; Risk Allele: rs3826656-?); (p-value= 0.000006). This variant is associated with Alzheimer's disease.
http://bio2rdf.org/dbsnp:rs3826656	CD33	In a GWAS of Alzheimer Disease families of self-reported European ancestry, this SNP was found to be associated with Alzheimer Disease. This association was also significant in replication attempts with three other family samples.
http://bio2rdf.org/dbsnp:rs2681411	CD86	This variant is significantly associated with late-onset Alzheimer disease (LOAD) in a GWAS study with 492 LOAD cases and 498 cognitive controls using Illumina's HumanHap550 beadchip.
http://bio2rdf.org/dbsnp:rs7537752	CSF1	This variant is nominally associated with late-onset Alzheimer disease (LOAD) in 2 GWAS studies.
http://bio2rdf.org/dbsnp:rs2180566	DEFB123	This variant is significantly associated with late-onset Alzheimer disease (LOAD) in a GWAS study with 492 LOAD cases and 498 cognitive controls using Illumina's HumanHap550 beadchip.
http://bio2rdf.org/dbsnp:rs12044355	DISC1	GWAS results: Genome-wide association study implicates a chromosome 12 risk locus for late-onset Alzheimer disease. (Initial Sample Size: 492 cases, 496 controls; Replication Sample Size: 238 cases, 220 controls); (Region: 1q42.2; Reported Gene(s): DISC1; Risk Allele: rs12044355-?); (p-value= 0.000009). This variant is associated with Alzheimer's disease.
http://bio2rdf.org/dbsnp:rs12044355	DISC1	This variant is significantly associated with late-onset Alzheimer disease (LOAD) in a GWAS study with 492 LOAD cases and 498 cognitive controls using Illumina's HumanHap550 beadchip.
http://bio2rdf.org/dbsnp:rs3740057	DNMBP	This variant is associated with Alzheimer dementia in the Belgian population
http://bio2rdf.org/dbsnp:rs10883421	DNMBP	This variant is associated with Alzheimer dementia in the Belgian population
http://bio2rdf.org/dbsnp:rs2373115	GAB2	GWAS Results: GAB2 alleles modify Alzheimer's risk in APOE epsilon4 carriers

		(Initial Sample Size: 446 cases, 290 controls; Replication Sample Size: 415 cases, 260 controls; Risk Allele: rs2373115-G).
http://bio2rdf.org/dbsnp:rs6428503	GBP2	This variant is nominally associated with late-onset Alzheimer disease (LOAD) in 2 GWAS studies.
http://bio2rdf.org/dbsnp:rs10922573	GBP2	This variant is nominally associated with late-onset Alzheimer disease (LOAD) in 2 GWAS studies.
http://bio2rdf.org/dbsnp:rs6942930	INTS1	This variant is significantly associated with late-onset Alzheimer disease (LOAD) in a GWAS study with 492 LOAD cases and 498 cognitive controls using Illumina's HumanHap550 beadchip.
http://bio2rdf.org/dbsnp:rs16934131	KCNMA1	This variant is nominally associated with late-onset Alzheimer disease (LOAD) in 2 GWAS studies.
http://bio2rdf.org/dbsnp:rs659628	KCTD12	This variant is significantly associated with late-onset Alzheimer disease (LOAD) in a GWAS study with 492 LOAD cases and 498 cognitive controls using Illumina's HumanHap550 beadchip.
http://bio2rdf.org/dbsnp:rs11754661	MTHFD1L	This variant is significantly associated with late-onset Alzheimer disease (LOAD) in a GWAS study with 492 LOAD cases and 498 cognitive controls using Illumina's HumanHap550 beadchip.
http://bio2rdf.org/dbsnp:rs11653716	NOS2	This variant is nominally associated with late-onset Alzheimer disease (LOAD) in 2 GWAS studies.
http://bio2rdf.org/dbsnp:rs2573905	PCDH11X	GWAS results: Genetic variation in PCDH11X is associated with susceptibility to late-onset Alzheimer's disease. (Initial Sample Size: 844 cases, 1,255 controls; Replication Sample Size: 1,547 cases, 1,209 controls); (Region: Xq21.31; Reported Gene(s): PCDH11X; Risk Allele: rs2573905-?); (p-value= 0.0000002). This variant is associated with Alzheimer's disease.
http://bio2rdf.org/dbsnp:rs3807031	PPP1R11	This variant is significantly associated with late-onset Alzheimer disease (LOAD) in a GWAS study with 492 LOAD cases and 498 cognitive controls using Illumina's HumanHap550 beadchip.
http://bio2rdf.org/dbsnp:rs6859	PVRL2	GWAS results: A genome-wide association study for late-onset Alzheimer's disease using DNA pooling. (Initial Sample Size: 1,082 cases, 1,239 controls; Replication Sample Size: 1,400 additional controls); (Region:

		19q13.32; Reported Gene(s): PVRL2, TOMM40, APOE; Risk Allele: rs6859-A); (p-value= 5.999999999999999E-14). This variant is associated with Alzheimer's disease.
http://bio2rdf.org/dbsnp:rs3846421	SORCS2	This variant is nominally associated with late-onset Alzheimer disease (LOAD) in 2 GWAS studies.
http://bio2rdf.org/dbsnp:rs10786828	SORCS3	This variant is nominally associated with late-onset Alzheimer disease (LOAD) in 2 GWAS studies.
http://bio2rdf.org/dbsnp:rs7894737	SORCS3	This variant is nominally associated with late-onset Alzheimer disease (LOAD) in 2 GWAS studies.
http://bio2rdf.org/dbsnp:rs720099	SORL1	This variant is nominally associated with late-onset Alzheimer disease (LOAD) in 2 GWAS studies.
http://bio2rdf.org/dbsnp:rs7946599	SORL1	This variant is nominally associated with late-onset Alzheimer disease (LOAD) in 2 GWAS studies.
http://bio2rdf.org/dbsnp:rs1784919	SORL1	This variant is nominally associated with late-onset Alzheimer disease (LOAD) in 2 GWAS studies.
http://bio2rdf.org/dbsnp:rs3781835	SORL1	This variant is nominally associated with late-onset Alzheimer disease (LOAD) in 2 GWAS studies.
http://bio2rdf.org/dbsnp:rs3781838	SORL1	This variant is nominally associated with late-onset Alzheimer disease (LOAD) in 2 GWAS studies.
http://bio2rdf.org/dbsnp:rs6589885	SORL1	This variant is nominally associated with late-onset Alzheimer disease (LOAD) in 2 GWAS studies.
http://bio2rdf.org/dbsnp:rs2298814	SORL1	This variant is nominally associated with late-onset Alzheimer disease (LOAD) in 2 GWAS studies.
http://bio2rdf.org/dbsnp:rs1792124	SORL1	This variant is nominally associated with late-onset Alzheimer disease (LOAD) in 2 GWAS studies.
http://bio2rdf.org/dbsnp:rs11218343	SORL1	This variant is nominally associated with late-onset Alzheimer disease (LOAD) in 2 GWAS studies.
http://bio2rdf.org/dbsnp:rs11218342	SORL1	This variant is nominally associated with late-onset Alzheimer disease (LOAD) in 2 GWAS studies.
http://bio2rdf.org/dbsnp:rs157580	TOMM40	GWAS results: Examination of the current top candidate genes for AD in a genome-wide association study. (Initial Sample Size: 491 cases, 479 controls; Replication Sample Size: NR); (Region: 19q13.32; Reported Gene(s): TOMM40, APOE; Risk Allele: rs157580-?);

Supplement 01 (v03) to "The Translational Medicine Ontology and Knowledge Base: Driving personalized medicine by bridging the gap between bench and bedside", Joanne S. Luciano et al., JBMS, Bio-Ontologies 2010 (2011)

		(p-value= 1E-40).This variant is associated with Alzheimer's disease.
http://bio2rdf.org/dbsnp:rs929156	TRIM15	This variant is significantly associated with late-onset Alzheimer disease (LOAD) in a GWAS study with 492 LOAD cases and 498 cognitive controls using Illumina's HumanHap550 beadchip.
http://bio2rdf.org/dbsnp:rs12514426	WWC1	This variant is nominally associated with late-onset Alzheimer disease (LOAD) in 2 GWAS studies.
http://bio2rdf.org/dbsnp:rs2061333	ZNF224	GWAS results: Genome-wide association study implicates a chromosome 12 risk locus for late-onset Alzheimer disease. (Initial Sample Size: 492 cases, 496 controls; Replication Sample Size: 238 cases, 220 controls); (Region: 19q13.31; Reported Gene(s): ZNF224; Risk Allele: rs2061333-?); (p-value= 0.000002).This variant is associated with Alzheimer's disease.
http://bio2rdf.org/dbsnp:rs4459653	ZNF224	This variant is significantly associated with late-onset Alzheimer disease (LOAD) in a GWAS study with 492 LOAD cases and 498 cognitive controls using Illumina's HumanHap550 beadchip.
http://bio2rdf.org/dbsnp:rs2061333	ZNF224	This variant is significantly associated with late-onset Alzheimer disease (LOAD) in a GWAS study with 492 LOAD cases and 498 cognitive controls using Illumina's HumanHap550 beadchip.
http://bio2rdf.org/dbsnp:rs2061332	ZNF224	This variant is significantly associated with late-onset Alzheimer disease (LOAD) in a GWAS study with 492 LOAD cases and 498 cognitive controls using Illumina's HumanHap550 beadchip.
http://bio2rdf.org/dbsnp:rs4802207	ZNF224	This variant is significantly associated with late-onset Alzheimer disease (LOAD) in a GWAS study with 492 LOAD cases and 498 cognitive controls using Illumina's HumanHap550 beadchip.
http://bio2rdf.org/dbsnp:rs3746319	ZNF224	This variant is significantly associated with late-onset Alzheimer disease (LOAD) in a GWAS study with 492 LOAD cases and 498 cognitive controls using Illumina's HumanHap550 beadchip.
http://bio2rdf.org/dbsnp:rs2061333	ZNF225	GWAS results: Genome-wide association study implicates a chromosome 12 risk locus for late-onset Alzheimer disease. (Initial Sample Size: 492 cases, 496 controls; Replication Sample Size: 238 cases, 220

		controls); (Region: 19q13.31; Reported Gene(s): ZNF224; Risk Allele: rs2061333-?); (p-value= 0.000002). This variant is associated with Alzheimer's disease.
http://bio2rdf.org/dbsnp:rs2061333	ZNF225	This variant is significantly associated with late-onset Alzheimer disease (LOAD) in a GWAS study with 492 LOAD cases and 498 cognitive controls using Illumina's HumanHap550 beadchip.
http://bio2rdf.org/dbsnp:rs2061332	ZNF225	This variant is significantly associated with late-onset Alzheimer disease (LOAD) in a GWAS study with 492 LOAD cases and 498 cognitive controls using Illumina's HumanHap550 beadchip.
http://bio2rdf.org/dbsnp:rs3746319	ZNF225	This variant is significantly associated with late-onset Alzheimer disease (LOAD) in a GWAS study with 492 LOAD cases and 498 cognitive controls using Illumina's HumanHap550 beadchip.
http://bio2rdf.org/dbsnp:rs3807031	ZNRD1	This variant is significantly associated with late-onset Alzheimer disease (LOAD) in a GWAS study with 492 LOAD cases and 498 cognitive controls using Illumina's HumanHap550 beadchip.

Q11. An APOE variant is strongly correlated with AD predisposition. Are there drug classes and drugs that target APOE?

SPARQL

PREFIX pharmgkb: <<http://bio2rdf.org/pharmgkb:>>

```

SELECT DISTINCT ?drug_name
WHERE {
  GRAPH <pharmgkb> {
    ?association rdf:type pharmgkb:DrugGeneVariantInteraction .
    ?association pharmgkb:description ?description .
    ?association pharmgkb:disease ?disease .
    ?association pharmgkb:variant ?variant .
    ?disease rdfs:label ?disease_name .
    FILTER regex(?disease_name, "alzheimer", "i") .
    ?association pharmgkb:gene ?gene .
    ?gene dc:identifier "APOE" .
    ?a2 a pharmgkb:Association .
    ?a2 pharmgkb:gene ?gene .
    ?a2 pharmgkb:disease ?d2 .
    ?d2 rdfs:label ?disease2_name .
    FILTER regex(?disease2_name, "alzheimer", "i") .
  }
}

```

Supplement 01 (v03) to "The Translational Medicine Ontology and Knowledge Base: Driving personalized medicine by bridging the gap between bench and bedside", Joanne S. Luciano et al., JBMS, Bio-Ontologies 2010 (2011)

```

        ?a2 pharmgkb:drug ?drug .
        ?drug rdfs:label ?drug_name .
    }
}
ORDER BY ASC(?drug_name)

```

Results

drug_name

Q12. Which existing marketed drugs might potentially be re-purposed for AD because they are known to modulate genes that are implicated in the disease?

PREFIX pharmgkb: <http://bio2rdf.org/pharmgkb:>

```

SELECT DISTINCT ?drug_name ?disease2_name
WHERE {
    GRAPH <pharmgkb> {
        ?association rdf:type pharmgkb:DrugGeneVariantInteraction .
        ?association pharmgkb:description ?description .
        ?association pharmgkb:disease
        <http://bio2rdf.org/pharmgkb:1aa122e7b0687fa04b895ee82d6b6476> .
        ?association pharmgkb:variant ?variant .
        ?association pharmgkb:gene ?gene .
        ?gene dc:identifier ?gene_name .
        ?a2 a pharmgkb:Association .
        ?a2 pharmgkb:gene ?gene .
        ?a2 pharmgkb:disease ?d2 .
        ?d2 rdfs:label ?disease2_name .
        ?a2 pharmgkb:drug ?drug .
        ?drug rdfs:label ?drug_name .
    }
}
ORDER BY ASC(?drug_name) ASC(?disease2_name)

```

Results

drug_name	disease2_name
(s)-rolipram	Autistic Disorder
(s)-rolipram	Bipolar Disorder
(s)-rolipram	Depression
(s)-rolipram	Schizophrenia
ACE INHIBITORS, PLAIN	Abnormalities
ACE INHIBITORS, PLAIN	Alcoholism
ACE INHIBITORS, PLAIN	Alzheimer Disease
ACE INHIBITORS, PLAIN	Angioneurotic Edema
ACE INHIBITORS, PLAIN	Cardiovascular Abnormalities

Supplement 01 (v03) to "The Translational Medicine Ontology and Knowledge Base: Driving personalized medicine by bridging the gap between bench and bedside", Joanne S. Luciano et al., JBMS, Bio-Ontologies 2010 (2011)

ACE INHIBITORS, PLAIN	Cardiovascular Diseases
ACE INHIBITORS, PLAIN	Coronary Disease
ACE INHIBITORS, PLAIN	Cough
ACE INHIBITORS, PLAIN	Fetal Death
ACE INHIBITORS, PLAIN	Heart Failure
ACE INHIBITORS, PLAIN	Hypertension
ACE INHIBITORS, PLAIN	Hypertrophy, Left Ventricular
ACE INHIBITORS, PLAIN	Kidney Diseases
ACE INHIBITORS, PLAIN	nondiabetic proteinuric nephropathy
ANGIOTENSIN II ANTAGONISTS AND<br>CALCIUM CHANNEL BLOCKERS	Cardiovascular Diseases
ANGIOTENSIN II ANTAGONISTS AND<br>CALCIUM CHANNEL BLOCKERS	Hypertension
ANTIPSYCHOTICS	Schizophrenia
BETA BLOCKING AGENTS	Abnormalities
BETA BLOCKING AGENTS	Cardiovascular Abnormalities
BETA BLOCKING AGENTS	Fetal Death
atenolol	Hypertension
atenolol	glomerulosclerosis
benazepril	Diabetes Mellitus, Non-Insulin-Dependent
benazepril	Hypertension
benazepril	Proteinuria
captopril	Hypertension
cilazapril	Cough
enalapril	Arteriosclerosis
enalapril	Hypertension
enalapril	glomerular disease
enalapril	glomerulosclerosis
epoetin alfa	Kidney Failure
ethanol	Alcoholism
fluvastatin	Arteriosclerosis
gemfibrozil	visceral obesity
hydrochlorothiazide	Hypertension
irbesartan	Hypertension
lisinopril	Diabetes Mellitus, Insulin-Dependent
lisinopril	Hypertension
nifedipine	Hypertension
perindopril	Cardiovascular Diseases
perindopril	Cerebrovascular Accident
perindopril	Dementia
perindopril	Diabetes Mellitus, Non-Insulin-Dependent
perindopril	Hypertension
perindopril	Memory Disorders
perindopril	Proteinuria
pravastatin	Coronary Arteriosclerosis

Q13. What are the results of Georg Steffen Möller's lipid panel?

SPARQL

PREFIX tmo: <tag:eric@w3.org:2009/tmo/translator#>

PREFIX foaf: <http://xmlns.com/foaf/0.1/>

SELECT ?date ?value ?units ?referrer ?referred_to ?clinic

```
WHERE {
    ?patient foaf:name "Georg Steffen Möller" .
    ?encounter tmo:patient ?patient ;
        tmo:dateOfEncounter ?date ;
        tmo:test ?referral .
    ?referral tmo:physicianNotes ?notes ;
        tmo:testType tmo:Laboratory ;
        tmo: testName "Lipid panel" ;
        tmo:CPT ?cpt ;
        tmo:referredTo ?referred_to ;
        tmo:referredClinic ?clinic ;
        tmo:referredBy ?referrer ;
        tmo:testSet ?panel .
    ?panel a tmo:LipidPanel ;
        tmo:testCode ?code .
    ?code tmo:description "Low-density lipoprotein" ;
        tmo:value ?value ;
        tmo:units ?units .
}
```

Results

date	value	units	referrer	referred_to	clinic
2008-05-06	4	mmol/l	David Winziski	Daniel Schmidt	Herzzentrum
2008-05-06	4	mmol/l	David Winziski	Daniel Schmidt	Herzzentrum

-----

Q14. What is Monica Mary Mall's platelet count over time?

SPARQL

PREFIX tmo: <tag:eric@w3.org:2009/tmo/translator#>

PREFIX foaf: <http://xmlns.com/foaf/0.1/>

SELECT ?date ?value ?units

```
WHERE {
    ?encounter tmo:patient [ foaf:name "Monica Mary Mall" ] ;
        tmo:dateOfEncounter ?date ;
        tmo:test [
```

Supplement 01 (v03) to "The Translational Medicine Ontology and Knowledge Base: Driving personalized medicine by bridging the gap between bench and bedside", Joanne S. Luciano et al., JBMS, Bio-Ontologies 2010 (2011)

```

    tmo:testSet [
      tmo:testCode [
        tmo:description "Platelet Count" ;
        tmo:value ?value ;
        tmo:units ?units
      ]
    ]
  ]
}
ORDER BY DESC(?date)

```

Results

date	value	units
2009-07-20	290	K/mcL
2008-07-20	290	K/mcL
2008-03-18	291	K/mcL
2007-07-18	291	K/mcL
2006-07-20	297	K/mcL
2005-07-20	291	K/mcL
2004-07-20	291	K/mcL
